# Electric field stimulation-responsive hydrogels for bone regeneration: from mechanisms to applications

**DOI:** 10.1038/s41413-025-00482-5

**Published:** 2026-01-12

**Authors:** Lizhi Ouyang, Xi He, Yuheng Liao, Xing Zhou, Jiewen Liao, Ze Lin, Xudong Xie, Weixian Hu, Wenqian Zhang, Fawwaz Al-Smadi, Ranyang Tao, Faqi Cao, Yiqiang Hu, Guohui Liu, Bobin Mi

**Affiliations:** 1 Department of Orthopedics, Union Hospital, Tongji Medical College, Huazhong University of Science and Technology, Wuhan, China; 2 Department of Rheumatology, Renji Hospital Affiliated to Shanghai Jiao Tong University School of Medicine, Shanghai, China; 3 MSk Laboratory, Sir Michael Uren Hub, Department of Surgery and Cancer, Faculty of Medicine, Imperial College London, London, UK

**Keywords:** Bone, Bone quality and biomechanics

## Abstract

The continuous extension of human life expectancy and the global trend of population aging have contributed to a marked increase in the incidence of musculoskeletal diseases, with fractures and osteoporosis being prominent examples. Consequently, promoting bone regeneration is a crucial medical challenge that demands immediate attention. As early as the mid-20th century, researchers revealed that electrical stimulation could effectively promote the healing and regeneration of bone tissue. This is achieved by mimicking the endogenous electric field within bone tissue, which influences cellular behavior and molecular mechanisms. In recent years, electroactive hydrogels responsive to electric field stimulation have been developed and applied to regulate cell functions at different stages of bone regeneration. This paper elaborates on the regulatory effects of electrical stimulation on MSCs, macrophages, and vascular endothelial cells during the process of bone regeneration. It also involves the activation of relevant ion channels and signaling pathways. Subsequently, it comprehensively reviews various electric-field-responsive hydrogels developed in recent years, covering aspects such as material selection, preparation methods, characteristics, and their applications in bone regeneration. Ultimately, it provides an objective summary of the existing deficiencies in hydrogel materials and research, and looks ahead to future development directions.

## Introduction

Bones are essential supporting organs in the human body. With the extension of life expectancy and the aging of the world’s population, musculoskeletal diseases and disorders such as fractures, osteoporosis, and bone metastases have increased rapidly. According to statistics in 2019, there were as many as 178 million fracture cases worldwide.^1^ Meanwhile, the occurrences of non-union fractures or delayed union of fractures have also been on the rise. Despite the adoption of standardized treatment measures, the incidence rates of these problems remain as high as 4.9%,^2^ which has seriously affected patients’ quality of life and caused a substantial socioeconomic burden. In addition, there is an equally urgent need for the repair of non-traumatic bone defects, such as bone reshaping surgery involving irregular incision shapes,^3^ and alveolar ridge reconstruction surgery requiring bone implants,^4^ all of which clearly demand advanced bone repair strategies.

As early as the middle of the last century, scientists discovered that applying electrical stimulation (ES) to both ends of bone in vitro could promote the healing and regeneration of bone tissue. In 1963, T. Cieszyński discovered the influence of bone conductivity on the human body’s healing ability and verified this finding in experimental animals.^5^ In 1970, he first used ES to treat 67 patients with delayed fractures, achieving sound curative effects.^6^ From 1970 to 2000, doctors and scientists from various countries further explored the impact of ES on fracture healing in clinical practice and animal models, and relevant modeling parameters were determined^7,8^ Moreover, a 10-year follow-up study was conducted on patients with delayed fracture healing treated with ES, demonstrating the safety and effectiveness of ES in promoting osteogenesis.^7^ This effect may be due to exogenous electrical stimulation simulating the endogenous electric field of bone. It has been reported that an electric field of a certain magnitude exists inside bone tissue. From the perspective of the whole bone, under normal circumstances, the epiphyseal region is electronegative, while the axial region is isopolar.^8^ From a cellular standpoint, the difference in ion concentrations inside and outside the cell constitutes a potential difference, and the electric field surrounds the endogenous cells (osteoblasts, osteoclasts, and osteocytes). When a fracture occurs, the electronegativity of the whole bone increases, with the metaphysis being the most electronegative.^8^ When electrons and anions accumulate at the fracture site, they carry a significant amount of negative charge, causing ions to flow to the injured site. Free ions move along the concentration gradient, resulting in chemical and electrical movements. When ions enter the surrounding cells, a local electric field of 1–2 V/cm is generated.^9^ The ion movement enters the damaged site as a current loop and exits from the intact bone upstream.^10^

Additionally, researchers have discovered that when bone tissue is compressed and deformed, electrical signals are generated at both ends of the bone, a phenomenon known as the piezoelectric effect. Scientists discovered and reported this effect in the mid-20th century. Specifically, the compressed bone surface can generate a negative potential, leading to osteogenesis. Meanwhile, the bone mass under the compressed surface generates a positive potential, resulting in bone resorption. The intensity of the generated electric field is directly related to the applied pressure.^11^ According to research reports, when a person walks, a direct current of approximately 300 mV is generated in the tibia.^8^ When the pressure applied to the bone approaches zero, the generated electric field also approaches zero. In this case, the bone will start to deteriorate.^12^ For a long time, it has been believed that the piezoelectric properties of bone originate from the non-centrosymmetric collagen, the main structural protein that forms bone, and the ion flow within the bone.^13,14^ However, theoretically, when any material undergoes non-uniform deformation, its electric dipole moment changes. Fabian et al. found through experiments that bending bone and hydroxyapatite can generate an electric field, inducing osteocyte apoptosis and initiating bone injury healing.^15^

In recent years, hydrogels, as a promising type of biomaterial, have been widely applied in the field of bone repair and regeneration. Due to their excellent biocompatibility and structural support capabilities, hydrogels serve as versatile drug carriers, enabling precise delivery of therapeutic agents to achieve multiple effects^16^—such as promoting osteogenesis, inhibiting osteoclast activity, and regulating immune responses—thereby offering significant advantages for disease treatment.^17–19^ With the increasing understanding of bone regeneration mechanisms, a growing number of studies have integrated ES with hydrogel-based polymeric biomaterials, resulting in the development of a series of electric field stimulation-responsive hydrogels. These hydrogels incorporate conductive, piezoelectric, triboelectric, or electrostatic functional components, endowing them with the ability to sense external electric fields or generate endogenous electrical signals. Through electro-biological interactions, they modulate the cellular behavior of MSCs, macrophages, and vascular endothelial cells, including proliferation, differentiation, migration, and intercellular signaling, while leveraging the 3D porous architecture and biocompatibility of hydrogels to create an electro-mechanical-biological synergistic microenvironment conducive to bone tissue regeneration.

**Fig. 1:**
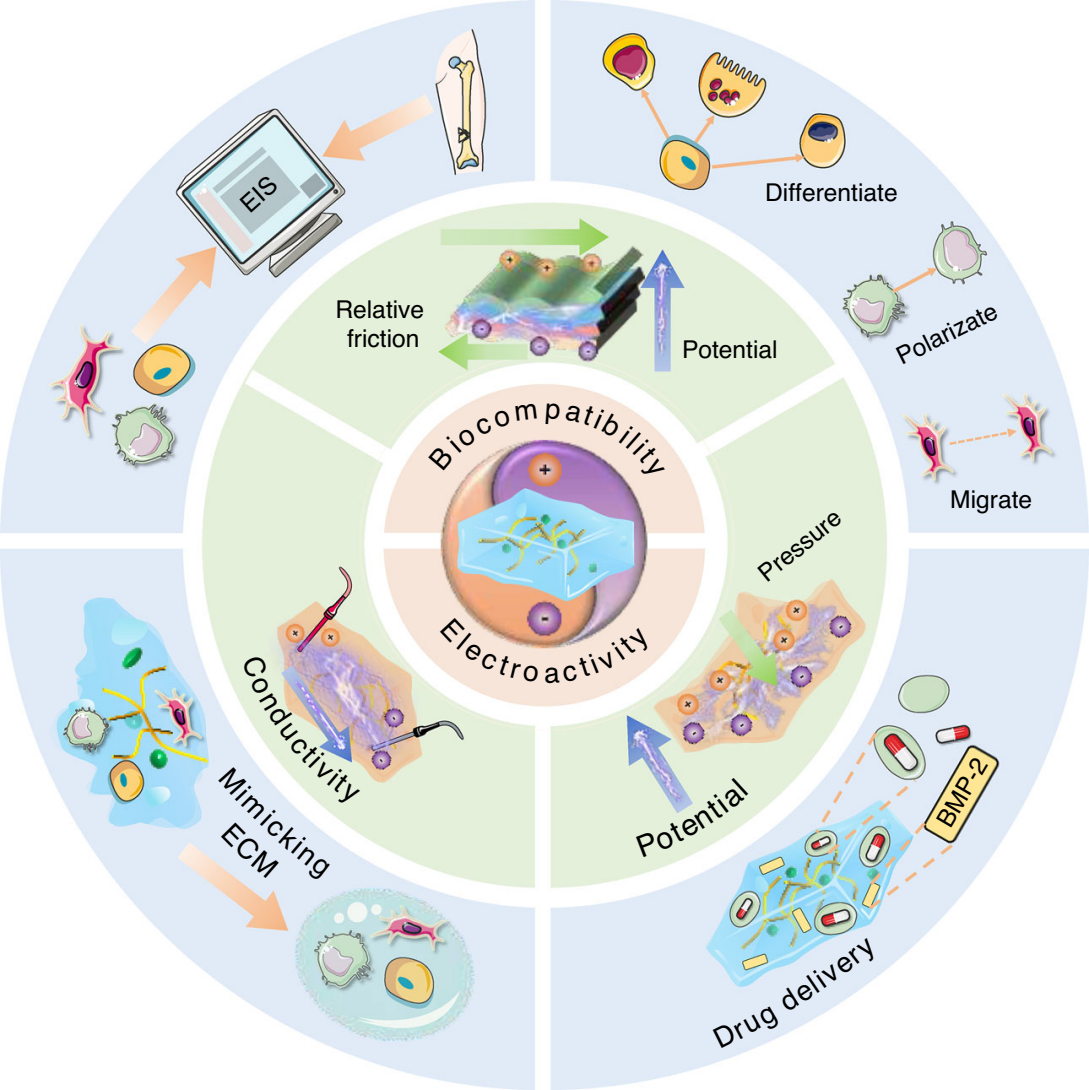
Graphic abstract of this article. This article mainly discusses the following three parts: (1) Cellular and molecular mechanisms related to bone regeneration; (2) Several electrical hydrogels responsive to electric field stimulation; (3) Biomedical applications of electrical hydrogels responsive to electric field stimulation

In this paper, we systematically investigate the pathophysiological mechanisms of bone regeneration, with a particular focus on the role of ES in this process. The article will then introduce various types of hydrogels with distinct electrical characteristics and their applications in bone repair. Finally, we look forward to future development trends and propose possible challenges (Fig. 1).

## Electrical stimulation promotes bone regeneration: cellular responses

**Fig. 2:**
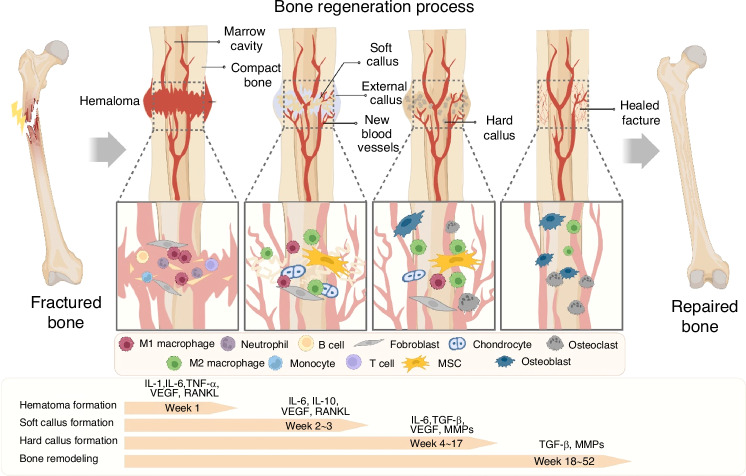
Bone regeneration is a complex and interconnected process that occurs in distinct phases. In the first phase (week 1), hematoma formation occurs, where the coagulation cascade triggers both pro-inflammatory and anti-inflammatory events, coordinated by IL-1, IL-6, TNF-α, VEGF, and RANKL, involving M1 and M2 macrophages, Th1 and Th2 cells, and fibroblasts. During week 2 to 3 soft callus formation and angiogenesis occur, involving endothelial cells, hypertrophic chondrocytes, and osteoblasts. During week 4−17 complex callus formation is characterized by matrix mineralization and woven bone development, with the participation of endothelial cells, osteocytes, osteoblasts, and osteoclasts. The bone remodeling phase (week 18–52) involves remodeling using TGF-β and MMPs, which affects endothelial cells, osteocytes, osteoblasts, and osteoclasts, thereby improving the healed fracture and restoring the functional bone structure. This complex temporal and cellular orchestration is the foundation for the successful regeneration of bone tissue. The figure here is based on the work of S. Park and Y. Niu et al. VEGF vascular endothelial growth factor, RANKL nuclear factor κB ligand, MMP matrix metalloproteinases.^227,228^ Copyright © 2024 Elsevier Ltd

The repair and regeneration of bone injuries involve complex and interrelated biological processes (Fig. 2). Bone regeneration mainly includes two pathways: intramembranous ossification and endochondral ossification.^20–22^ Intramembranous ossification usually occurs in flat bones such as the skull. Mesenchymal stem cells (MSCs) directly differentiate into osteoblasts and deposit a mineralized extracellular matrix (ECM) during this process. In addition, MSCs also possess extensive immunomodulatory properties, making them a crucial target in tissue engineering.^23^ In fractures of long bones, due to poor mechanical stability and a relatively large fracture gap, healing needs to go through the classic stages of endochondral ossification, which can be mainly divided into four stages^24^:

The first stage is also known as the inflammatory phase. Neutrophils are an essential line of defense for the body against infections. They respond rapidly in the early stage of inflammation and release inflammatory mediators. When a fracture occurs, neutrophils are the first to arrive at the fracture hematoma and are quickly infiltrated by immune cells such as macrophages and neutrophils. They clear necrotic tissues and provide a signaling environment for subsequent stages.^25^ After 24–48 h, monocytes are recruited and migrate to the hematoma, differentiate into macrophages, and secrete growth factors and cytokines to play their pro-inflammatory roles.^26^ Macrophages have a strong phagocytic ability. In tissue repair, they participate in the inflammatory response and can regulate processes such as ECM remodeling. Meanwhile, platelets and red blood cells form an initial clot to reduce bleeding and provide a temporary framework for subsequent remodeling. In addition to their clotting function, platelets can release growth factors that promote cell proliferation and tissue repair. MSCs in the periosteum proliferate and differentiate into osteogenic and/or chondrogenic cells.^27^

In the second stage, new blood vessels are formed, and cartilage formation proceeds through two stages: cartilage callus formation and bony callus formation.^24^ During cartilage callus formation, MSCs respond to local signals and differentiate into chondrocytes, which actively secrete a cartilage matrix containing type II collagen. These matrices gradually accumulate to form a cartilage callus, initially filling the fracture gap and providing a certain degree of stability. Then, during the process of bony callus formation, some MSCs transform into osteoblasts, synthesize bone matrix, such as type I collagen, and mineralize it, thereby forming a bony callus around the cartilage callus. This enhances the stability of the fracture site, further strengthening the structural basis for subsequent bone remodeling and effectively promoting the continuous and orderly progression of bone repair.^28^

The third stage is the remodeling phase. Cartilage gradually expands and begins to calcify. At this stage, osteoclasts are gradually activated, and osteogenesis and osteoclastogenesis occur in an alternating manner. Osteoclasts are multinucleated giant cells formed by the fusion of mononuclear macrophage precursors. Their primary function is to absorb calcium and other minerals from the bone. They play a key role in bone remodeling, creating space for new bone formation by degrading old bone and regulating the balance of calcium and phosphorus.^29^

The fourth stage begins approximately three weeks after the fracture occurs and may last for several months to several years; therefore, it is referred to as the chronic phase.^30^ In this stage, woven bone is gradually replaced by lamellar bone. Osteoblasts remain active during this process. They deposit calcium and phosphorus ions into the bone matrix to promote mineralization, making the structure of the woven bone denser and more stable. Then, the woven bone is transformed into lamellar bone.^29^ Some osteoblasts become embedded and transform into osteocytes. Osteocytes form a network with surrounding cells through their dendritic protrusions. They sense mechanical stress and convert it into chemical signals to regulate the activities of osteoblasts and osteoclasts, maintaining the stability of the internal environment of bone tissue. Meanwhile, the overall size of the callus decreases, and the typical hematopoietic and trabecular structures are restored.

In this section, we will introduce three types of cells that play essential roles in bone regeneration: bone marrow-derived mesenchymal stem cells (BMSC), macrophages, and vascular endothelial cells, and elaborate on the impacts of electrical stimulation on them.

### Mesenchymal stem cells: the core in bone regeneration

BMSCs refer to non-hematopoietic, non-epithelial, and non-neuronal cells that support hematopoiesis and are found within the bone marrow. This term generally represents diverse adherent cell populations obtained from various connective tissues. In a narrow sense, it does not entirely refer to the stem cells in the bone marrow that can differentiate into osteoblasts and chondrocytes.^31^ However, in many studies, including this paper, for the sake of convenience, this term is often used to refer to the stem cells of chondrocytes, osteoblasts, and adipocytes in the bone marrow.

During the process of bone regeneration, BMSCs exhibit diverse roles and functions. In the process of fracture repair, BMSCs directly differentiate into osteoblasts or chondrocytes and form bone via endochondral ossification or intramembranous ossification. The differentiation depends on different signals and environments. The Wnt/β-catenin signaling pathway is a crucial factor that inhibits the differentiation of BMSCs into adipocytes and chondrocytes while promoting their differentiation into osteoblasts.^32^ Bone morphogenetic proteins (BMPs) comprise highly conserved soluble bone matrix glycoproteins with similar structures. An appropriate amount of BMPs can promote osteogenesis at the site of bone defects, increase the success rate of non-union fractures, promote angiogenesis at the bone repair site, and reduce complications such as fracture-related infections and pain.^33,34^ In addition, signaling molecules such as transforming growth factor beta (TGF-β), hedgehog pathways, fibroblast growth factors, and parathyroid hormone are essential signaling and effector molecules for regulating BMSCs.^35,36^ Additionally, environmental factors, metabolism, mechanical stress, hormones, and epigenetics can all influence cell differentiation. In the remodeling stages of bone repair, BMSCs differentia transforming growth factor betate into functionally mature osteoblasts. Osteoblasts synthesize and secrete large amounts of bone matrix proteins, such as type I collagen, osteopontin, and osteocalcin, promoting the nucleation and growth of hydroxyapatite crystals. Under the action of alkaline phosphatase (ALP), they encourage the formation of matrix vesicles and initiate the mineralization process. They also interact with osteoclasts to regulate the dynamic balance of mineralization.^37^

In addition to being the primary source of osteoblasts and chondrocytes, some scholars also utilize BMSCs to treat nerve injuries. Innervation is a key initiating factor for bone repair, playing a crucial regulatory role in the subsequent processes of vascularization, ossification, and mineralization. BMSCs repair nerve injuries primarily through two pathways: First, they can differentiate into nerve cells. Transplanting BMSCs into different physiological microenvironments can induce them to differentiate into neurons, astrocytes, and SC-like cells and express corresponding antigen markers.^38,39^ Second, they can synthesize and release proteins related to myelination and axon regeneration, such as brain-derived neurotrophic factor, nerve growth factor, vascular endothelial growth factor (VEGF), basic fibroblast growth factor, and insulin-like growth factor, thereby promoting the repair and regeneration of neurons.^40,41^

**Fig. 3:**
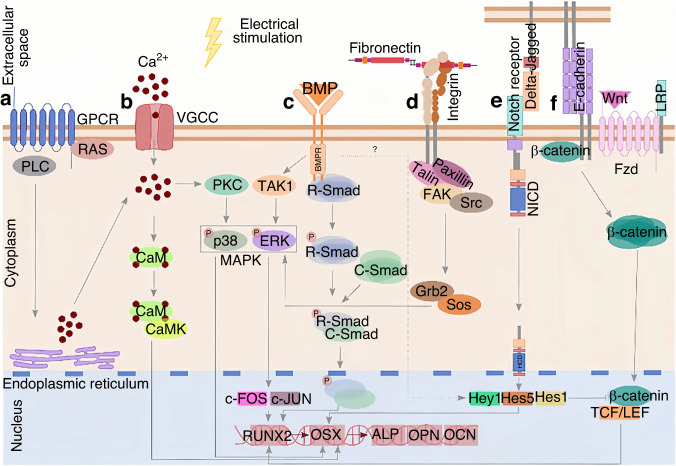
Electrical stimulation activates signaling pathways in BMSCs. **a** G-protein-coupled receptors bind PLC, releasing Ca^2+^ from the endoplasmic reticulum. The increase in intracellular calcium concentration activates protein kinase C, which in turn activates the MAPK pathway. **b** VGCC allows calcium to enter the cytoplasm, bind to CaM, interact with CaMK, and promote OSX expression. **c** Bone morphogenetic protein receptors can be activated by a combination of BMP ligands and electrical stimulation, activating the Smad-dependent pathway, leading to RUNX2 expression, or by activating the MAPK extracellular signal-regulated kinase (ERK) and p38 in the activation-independent pathway, which in turn can induce RUNX2 and OSX expression. **d** Piezoelectric substrates with associated surface potentials can enhance protein adsorption and change their conformation, exposing adhesion domains recognized by integrins. Integrins mediate the response by activating FAK. **e** Notch signaling pathway may be activated by electrical stimulation. When the NICD is cleaved and enters the nucleus, it promotes the expression of Hey1, Hes5, and Hes1. **f** Cell-cell junctions and piezoelectric stimulation can activate the Wnt/β-catenin signaling pathway. Due to the decrease in intracellular calcium concentration, β-catenin is released from E-cadherin, accumulating in the cytoplasm and translocating to the nucleus, promoting TCF/LEF expression. PLC phospholipase C, PKC protein kinase C, MAPK mitogen-activated protein kinase, VGCC voltage-gated calcium channels, CaM calmodulin, CaMK calmodulin-dependent protein kinase, OSX osterix, BMP bone morphogenetic protein, RUNX2 runt-related transcription factor, FAK focal adhesion kinase, NICD notch intracellular domain, TCF/LEF T cell factor/lymphoid enhancer factor.^44^ Copyright © 2024 Elsevier Ltd

In recent years, researchers have been interested in the effect of ES on the differentiation of BMSCs. Barker’s group used platinum electrodes coupled to the covers of six-well plates to stimulate the chambers and study the impact of direct current on the differentiation of BMSCs. They found that direct current-coupled ES (1 h per day) at 100 V/m combined with the osteogenic medium could upregulate genes related to osteogenic differentiation. The upregulation of calmodulin indicated that the calcium/calmodulin pathway was involved, and this effect was even more pronounced when combined with osteoinductive scaffolds.^42,43^ Figure 3 summarizes the process of electrical stimulation activating the downstream pathways of BMSCs.

Based on the effect of electrical stimulation on BMSCs, researchers have developed various methods and devices to stimulate these cells. Inductive coupling is one of the most commonly used methods to stimulate BMSCs. Induction coupling stimulation is based on the induction of an electric field by conductive coils or solenoids, thereby avoiding direct contact between the battery and electrodes, as well as by-products. There is no consensus on the optimal stimulation conditions for BMSCs, and parameters such as different magnetic field densities have been applied.^44^ Many scholars have found that combining pulsed electromagnetic fields with osteogenic matrices can increase the expression of early osteogenic markers.^45,46^ Martini et al. added BMP-2 and demonstrated the additive effect generated by simultaneously activating the Smad 1/5/8 and p38 MAPK pathways.^47^ However, Schwartz’s results showed that changes in the cell culture surface could affect the sensitivity of BMSCs to BMP-2 and pulsed electromagnetic fields, supporting the hypothesis that PEMF can affect the osteogenic differentiation of MSCs.^48^ Hess et al. developed a transformer-coupled device that can apply ES without magnetic field interference. They confirmed that PEMF must be combined with osteogenic medium to induce osteogenic differentiation. BMSCs cultured with highly sulfated hyaluronic acid derivatives can effectively deliver growth factors and act as BMP-2.^49,50^ Xiaowen Sun’s team fabricated positively charged 50 µm micro-pillars on a piezoelectric barium titanate oxide (BTO) substrate. The topological structure of this 50 µm micro-pillar array can generate the maximum surface electric field intensity and the highest residual polarization intensity. It can produce the best three-dimensional spatial ES. This biomimetic three-dimensional spatial electrical microenvironment can promote cell spreading, cytoskeleton reorganization, focal adhesion maturation, and osteogenic differentiation of BMSCs by amplifying integrin α5β1-mediated cell mechanosensing. When implanted into a rat femoral shaft defect model, the topographical pattern of the electropositive 50 µm micro-pillar array can establish a favorable three-dimensional spatial electrical microenvironment with the endogenous electronegative bone defect wall, accelerating bone integration in vivo.^51^

### Monocytes-Macrophages: The Balancer and Coordinator in Bone Regeneration

Macrophages and osteoclasts belong to the same mononuclear macrophage system and exhibit lineage relationships in bone repair. In the early stages of fracture healing, monocytes in the blood migrate to the injury site, with some differentiating into pro-inflammatory M1-type macrophages, which secrete cytokines such as TNF-α and IL-1β to recruit immune cells and clear necrotic tissue^52^; others, under the influence of local microenvironmental signals (e.g., receptor activator of nuclear factor kappa-B ligand (RANKL) and M-CSF), undergo directed differentiation into osteoclast precursors. As inflammation subsides, macrophages polarize toward the M2-type, participating in tissue repair and remodeling.^53^ Specifically, M2-type macrophages secrete anti-inflammatory factors such as TGF-β and IL-10, regulate the inflammatory response, promote angiogenesis, and stimulate the recruitment and differentiation of BMSCs and osteoblasts. During the remodeling phase, macrophages exhibit a mixed M1/M2 phenotype,^54^ while osteoclasts (derived from hematopoietic monocytes) initiate the bone resorption process. Osteoclasts, as multinucleated giant cells, strictly depend on RANKL secreted by osteoblasts for their differentiation, and they degrade necrotic bone matrix to provide space for new bone formation.^55^ Although macrophages and osteoclasts originate from different differentiation pathways of monocytes, they form a “inflammatory regulation-bone resorption” synergistic axis through a cytokine network, jointly driving the temporal progression of fracture repair.

During the fracture healing process, macrophages and BMSCs coordinate and interact with each other. BMSCs can be activated by the inflammatory factor interferon (IFN)-γ and three other co-existing pro-inflammatory cytokines (TNF-α, IL-1α, IL-1β).^56^ In an inflammatory microenvironment, MSCs increase the secretion of IL-10 by macrophages through inducible nitric oxide synthase- and Costal2-dependent pathways, inhibit the activation of inflammatory macrophages, and induce the polarization of macrophages into the M2 type.^57^ MSCs can also secrete numerous chemokines, including CC-motif chemokine ligand 2 (CCL2) and CCL4, to recruit monocytes and macrophages, thereby promoting tissue regeneration.^58,59^ While osteoclast-derived factors (e.g., TGF-β released from matrix degradation) reciprocally enhance BMSC osteogenic differentiation.^60^ This tripartite interaction—macrophages regulating inflammation, osteoclasts mediating resorption, and BMSCs driving bone formation—ensures balanced remodeling. Dysregulation, such as excessive osteoclast activity in osteoporosis, can impair repair efficiency.^61^

Electric field stimulation can promote the migration and polarization of macrophages. M. R. Cho et al. found that when macrophages were exposed to an electric field with a frequency of 1 Hz and an intensity of 2 V/cm, the induced migration speed on a glass substrate was (5.2 ± 0.4) × 10² μm/min, and the random motility coefficient was (4.8 ± 1.4) × 10² μm/min. Moreover, the electric field-induced macrophage migration employs multiple motility patterns, including cell crawling and possibly cell rolling.^62^ During the wound healing study, Junwei Xu’s team applied non-contact electrical stimulation to cells and mice and observed that ES promoted the M2 polarization of macrophages.^63^ Ying Kong’s team proposed a method to regulate macrophage polarization using local electrical signals generated by piezoelectric β-phase polyvinylidene fluoride (β-PVDF) films in a wireless mode. Ultrasound irradiation drives the release of charges on the surface of the β-PVDF film, which can enhance the polarization of M1-type macrophages.^64^ In the research on bone regeneration, the effectiveness of activating macrophages while inducing osteogenesis is a common focus among researchers. For this reason, when developing materials, researchers pay great attention to the polarization ability of macrophages. For example, Hao Wu’s team prepared a uniform BaTiO₃ layer piezoelectric BaTiO₃/Ti6Al4V (BT/Ti) scaffold by the hydrothermal method. The piezoelectric effect of this polarized BT/Ti scaffold can simultaneously promote the M2 polarization of macrophages and the immunomodulatory osteogenesis of MC-3T3 osteoblasts.^65^ Huifan Liu’s team developed a biomimetic periosteum with excellent piezoelectric effect, which can not only promote the adhesion, proliferation, spreading, and osteogenesis of MSCs but also effectively induce the polarization of M2 macrophages, thereby inhibiting the inflammatory response induced by reactive oxygen species (ROS).^66^

### Vascular endothelial cells: a powerful transportation network

Angiogenesis constitutes a multifunctional transport network that is critical for tissue oxygenation, metabolism, and immune surveillance. Vascular invasion is a key step in skeletal development, as blood vessels provide nutrients and oxygen to local tissues, remove metabolic waste, and supply signaling molecules necessary for osteogenesis.

In skeletal development, taking endochondral ossification as an example, hypertrophic chondrocytes in the growth plate secrete VEGF-A (a key regulatory factor in angiogenesis),^67^ which activates VEGFR2 on endothelial cells, promoting vascular invasion into the cartilage matrix. This process is closely linked to ossification and the formation of primary and secondary ossification centers. Concurrently, endothelial cells interact with the surrounding cartilage cell matrix via short filopodia, which guide vascular growth and branching.^68^ In this process, H-type endothelial cells (highly expressing CD31 and Emcn) are closely associated with osteoprogenitor cells and play a key role in bone formation; embryonic E-type endothelial cells can induce mesenchymal cells to differentiate into osteoblasts and further differentiate into H-type and L-type endothelial cells.^69^

Bone repair and bone development share the same regulatory logic for angiogenesis, but there are stage-specific differences. In the early inflammatory stage, osteoblast precursors are a crucial source of VEGF-A, which activates vascular endothelial growth factor receptor 2 (VEGFR2) on endothelial cells, thereby recruiting macrophages and initiating the repair process.^70^ During the endochondral ossification stage, osteoblasts and proliferative chondrocytes secrete VEGF-A, which stimulates vascular growth, recruits osteoclasts, and promotes cartilage resorption. During the remodeling stage, osteoblasts continue to secrete VEGF-A, thereby supporting the synergistic remodeling of both the vascular network and bone tissue.^71^

In addition to the VEGFA core pathway, Notch signaling regulates H-type endothelial cell differentiation,^72^ and hypoxia-inducible factor (HIF)-1α activates the coupling of angiogenesis and osteogenesis in hypoxic microenvironments.^73^ Some ligands of BMPs activate the endothelial cell angiogenesis program through the Smad pathway.^74,75^ Soluble slit-guided ligand 3 (SLIT3)-osteoblasts secrete ROBO signaling and regulate angiogenesis via a paracrine mechanism.^76,77^

Angiogenesis and osteoclast lineage interactively regulate each other. Osteoclasts induce H-type angiogenesis by secreting PDGF-B. In an ovariectomy model, reduced PDGF-B levels result in a decrease in H-type vessels, and exogenous supplementation can restore angiogenesis and bone mass.^78^ Osteoclast-derived MMP9 participates in vascular basement membrane remodeling, while MMP9 provided by vascular endothelial cells is more critical for cartilage resorption.^79^ Conversely, VEGF regulates bone resorption by inhibiting osteoclast motility.^80^ Osteocytes secrete VEGF through the lacuno-canalicular network in response to mechanical loading to stimulate H-type angiogenesis. In limb unloading models, a reduction in H-type capillaries is associated with bone loss, indicating that the mechanical homeostasis regulation between osteocytes and blood vessels.^81^

Electrical field stimulation can promote the proliferation and migration of vascular endothelial cells in multiple ways. This process is associated with the mechanosensitive Piezo1 channel in human umbilical vein endothelial cells (HUVECs). In the presence of an electric field, calcium ion polarization stimulates HUVECs to contract their cytoskeleton, activate the Piezo1 channel, and induce calcium ion influx from the extracellular space.^82^ This calcium signaling pathway further activates the PI3K/AKT and Erk1/2 signaling pathways. For example, Wang et al. used high-voltage unidirectional pulse currents to promote the proliferation and migration of HUVECs in diabetic wound healing.^83^ Lu’s team confirmed that electric field stimulation can induce HUVECs to elongate, migrate, and form connections in cardiac tissue, while enhancing the secretion of signaling factors that interact between cardiomyocytes and HUVECs.^84^

Different electric stimulation parameters exhibit differentiated regulatory effects: appropriate electrical pulses (5 V, 5 Hz, 6 ms) can enhance the adhesion function between endothelial cells and progenitor cells^85^; while low-frequency short pulses (0.5 Hz, 0.5 ms) induce contraction of the arterial intima and relaxation of the adventitia, leading to a reduction in vessel diameter.^86^ Based on this, the Mao team constructed an electro-mechanical coupling feedback loop for HUVECs—when cells are cultured on patterned piezoelectric materials, an exogenous electric field (*E*_ex_) induces intracellular calcium ion ([Ca^2+^]i) polarization, forming an intrinsic electric field (*E*_in_) opposite to Eex; the polarization of [Ca^2+^]i further stimulates cytoskeletal contraction, promoting calcium influx through Piezo1 channels, gradually balancing Ein with Eex. This dynamic coupling process activates calcium dynamics to regulate the eNOS/NO angiogenesis pathway, guiding pre-angiogenic activities such as HUVEC alignment, elongation, and migration.^82^

At the clinical translation level, functional electrical stimulation not only improves exercise capacity and central hemodynamics in patients with chronic heart failure but also serves as an alternative treatment option for patients with exercise limitations by promoting angiogenesis and enhancing peripheral muscle endothelial function.^87^ These findings provide a comprehensive evidence chain from cellular mechanisms to clinical applications for electrical stimulation-mediated vascular regeneration therapy.

## Electrical stimulation promotes bone regeneration: ion channels

The endogenous electric field of cells constitutes the basis of all cellular physiological processes. The difference in ion concentrations inside and outside the cell generates an electric field on the cell membrane called the membrane potential. Charged ions achieve internal and external ion exchange through ion pumps, channels, or gap junctions. Changes in ion balance and membrane potential may lead to the activation of voltage-gated ion channels on the cell membrane. This alters the conformation of membrane proteins and the movement and concentration of molecules on the cell membrane and within the cell.^88^ Excitable cells can generate action potentials, which are crucial for communication between neurons and the contraction of skeletal and cardiac muscles. Non-excitable cells can also respond to changes in membrane potential, including proliferation, orientation, migration, differentiation, and apoptosis. These responses involve biological processes such as circadian rhythms, biosensing, cell volume regulation, wound healing, and regeneration.^89^ Among numerous mechanisms, ion channels are particularly crucial. Different types of gated ion channels perform their respective functions. For example, voltage-gated ion channels generate action potentials, whereas ligand-gated ion channels facilitate neuronal signal transmission.^90^ In research on osteogenesis and development, we focus on ion channels, including voltage-gated calcium channels, piezoelectric channels, and transient receptor potential (TRP) channels. In the microenvironment of bone tissue, the changes in ion channels and the activation of signal pathways induced by ES are closely intertwined. Its unique electrical properties are closely interwoven with the physiological activities of bone cells. They can accurately and deeply reflect the key electrical regulatory mechanisms in bone development.^90–92^ Therefore, in this part, we have selected several of the most essential ion channels in the process of osteogenesis and development, including voltage-gated calcium channels, piezoelectric channels, TRP channels, and elaborated on the changes in ion channels and the activation of signal pathways triggered by ES from a more microscopic perspective.

### Voltage-gated channels

When electrical signals or mechanical loads stimulate bones, L-type voltage-sensitive calcium channels (CaV1.2) activate, mediating Ca^2+^ influx into osteoblasts and driving osteogenic differentiation through multiple pathways. On the one hand, they activate the TGF-β1/Smad and Wnt/β-catenin signaling axes, promoting the expression of osteogenic transcription factors such as Runx2.^93,94^ On the other hand, they trigger calcium transients, regulating osteoprotegerin expression and activating the MAPK/PKC signaling network.^95–98^ Blocking CaV1.2 inhibits the differentiation of MSCs into the osteogenic lineage and osteoblast proliferation.^99–102^ Its reduced expression is a key driver of age-related bone loss.^103^ —Geoff Rey et al. discovered through CaV1.2 inactivating mutants that channel dysfunction leads to suppression of osteogenic transcription programs and impaired mineralization capacity.^104^ Jin’s team confirmed in genetically edited mice that downregulation of CaV1.2 expression reduces osteogenic capacity, while the calcium channel agonist Bay K 8644 can reverse this defect by restoring Ca^2+^ influx.^103^

In osteoclasts, electrical stimulation may inhibit footplate formation and bone resorption function through voltage-sensitive calcium channels (VSCCs).^105^ However, the primary regulatory mechanism stems from paracrine signals from osteoblasts—OPG released upon CaV1.2 activation can inhibit RANKL-induced osteoclast differentiation. At the same time, NO suppresses osteoclast bone resorption activity via the cGMP pathway.^106–109^ Recent studies have found that the voltage-gated potassium channel-related long non-coding RNA, KCNQ1OT1, is downregulated in osteoclasts in osteoporotic bone tissue. It relieves the inhibition of NFAT5 by sponge adsorption of miR-128-3p, thereby promoting osteoclast differentiation.^110^ During osteoblast differentiation into osteocytes, VSCCs transition from CaV1.2 to T-type channels (CaV3.2/CaV3.1), maintaining mechanical sensitivity through low-voltage activation characteristics. These channels mediate calcium signaling and ATP release induced by fluid shear stress, and this transition in channel type is critical for the long-term survival of osteocytes and the mechanical transduction.^111–113^

Among the novel materials developed for VSCC, the self-promoting electroactive mineralization scaffold (sp-EMS) developed by Liu et al. generates a weak current through spontaneous electrochemical reactions, activates voltage-gated Ca^2+^ channels, enhances ATP-induced actin remodeling, and promotes osteogenic differentiation of MSCs by activating the BMP2/Smad5 pathway.^114^ Photogenerated charges form a potential on the graphene-transfer-silicon (Gr/Si) surface, activating voltage-gated calcium channels, which leads to cytoplasmic Ca^2+^ accumulation and stimulates the osteogenic differentiation of BMSCs.^115^

Voltage-gated sodium channels (VGSCs) are present in various non-excitable cells. Low-intensity near-infrared light may promote bone regeneration through the Scn4a subunit of VGSCs in a manner dependent on the core circadian protein CRY1.^116^ Mutations in the voltage-gated chloride channel-7 (ClC-7) can lead to osteosclerosis. Research has shown that ClC-7 plays a crucial role in craniofacial bone and tooth development and mineralization in zebrafish, influencing craniofacial bone and tooth changes in patients with osteosclerosis and dense bone dysplasia through the ClC-7/CTSK/TGF-β/BMP/SMAD signaling pathway.^117^

### Piezoelectric channels

Piezoelectric channels are ion channels that are activated mechanically, enabling cells to detect mechanical forces. Piezo1 and Piezo2 channels are important mechanosensitive channels that play significant roles in skeletal development and bone homeostasis, regulating relevant signaling pathways and promoting osteoblast differentiation.^118^ Piezo1 exists in developing long bones and is expressed in connective tissues, related muscles, differentiated osteoblasts, hypertrophic chondrocytes, tendons, and ligaments^119^; Piezo2 is expressed in cells of formed fingers and wrist joints, some osteoblasts, growth plate chondrocytes, tendon, and connective tissue cells, suggesting that they may be involved in the mechanotransduction process during bone development.^120^ Currently, the functional characteristics of Piezo1 are widely recognized.

During bone regeneration, Piezo1 channels can regulate the fate of MSCs, thereby triggering osteoblast differentiation and inhibiting adipogenesis. By activating Piezo1, such as by applying fluid shear force or using its agonist Yoda1, genes related to osteogenesis, including those involved in the ERK1/2 and p38 signaling pathways associated with BMP2, can be upregulated, thereby promoting the osteogenic differentiation process of BMSCs.^121^ This regulatory role is significant for maintaining the normal development and repair of bones. Force-induced conformational changes can directly activate Piezo1, making it an essential mechanical force sensor in osteoblasts. In mouse embryonic development, Piezo1 plays a role in the osteogenic process, and its deletion affects bone formation.^120^ Additionally, Piezo1 collaborates with other ion channels, such as transient receptor potential vanilloid 4 (TRPV4), to regulate the proliferation and function of osteoblasts. In some cases, such as under the action of fluid shear force, the synergistic effect of Piezo1 and TRPV4 is crucial for regulating the calcium ion concentration in osteoblasts, which in turn affects the physiological state of the cells.^122^

Piezo1 plays a role in growth plate chondrocytes, contributing to chondrogenesis and endochondral ossification during the developmental process. After Piezo1 is activated, the cytoplasm’s calcium influx increases to protect articular chondrocytes from mechanically induced cell death.^123,124^ The selective Piezo1 agonist Yoda1 has been found to increase the Ca^2+^ level in human chondrocytes with osteoarthritis and upregulate the expression of MMPs, tissue inhibitor of metalloproteinase 2, BMP2, type I collagen α1, and interleukin.^125^ Blocking the Piezo1/CaN/NFAT1 signaling axis can protect chondrocytes from apoptosis and anabolic/catabolic imbalance under mechanical strain. For example, GsMTx4 can specifically block the signal transduction of this signaling axis, reduce intracellular calcium concentration, inhibit the nuclear translocation of nuclear factor of activated T cells 1 (NFAT1), and protect chondrocytes from apoptosis, as well as anabolic and catabolic imbalance.^126^ In osteocytes, Piezo1 is an abundantly expressed ion channel crucial for the transduction of mechanical signals. During mechanical loading, osteocytes sense fluid flow through Piezo1 and convert mechanical signals into intracellular biochemical signals, regulating the expression of relevant genes and cell functions. For example, the Piezo1-Akt signaling pathway plays a role in the mechanotransduction of osteocytes. Inhibiting the expression of sclerostin promotes bone formation.^127^ In addition, Piezo1 interacts with other molecules, such as primary cilia, and jointly participates in the response of osteocytes to mechanical stimuli, maintaining the homeostatic balance of bones. When the function of Piezo1 is impaired, the ability of osteocytes to respond to mechanical forces decreases, which may lead to abnormal adaptive remodeling of bones and affect the health and function of bones.^128^

### Transient receptor potential channels

TRP channels are a class of cation channels. According to sequence homology, they can be divided into seven subfamilies (TRPC, TRMPV, TRPM, TRPA, TRPL, TRPP, and TRPV). They comprise six transmembrane helices and are usually organized as functionally homologous or heterotetramers. Many stressors can regulate or cause them, including heat, pressure, tension, shear stress, oxidative stress, and hypoxia.^129^

TRP channels are widely present in osteoblasts. Current studies have identified multiple TRP channels in osteoblasts, including TRPC1, 3, 4, and 6, as well as TRPV2, 4, and TRPM4, 6, 7, and 8, among others.^130,131^ Among them, TRPV4 can regulate the expression of RANKL and NFATc1 in coordination with TRPM3, thereby affecting the differentiation of osteoblasts.^132^ TRPV1 has a bidirectional effect on osteoblasts. TRPV1 can inhibit the differentiation of BMSCs into osteoblasts through a mechanism involving TNF-α and other inflammatory factors. However, low-level laser irradiation stimulation of TRPV1 can promote the proliferation of osteoblasts and increase the expression of osteogenic markers such as Runx2, Osx, Alp, and Opn.^133^ The selective inhibitor capsazepine can reverse this promoting effect.^134^

**Fig. 4:**
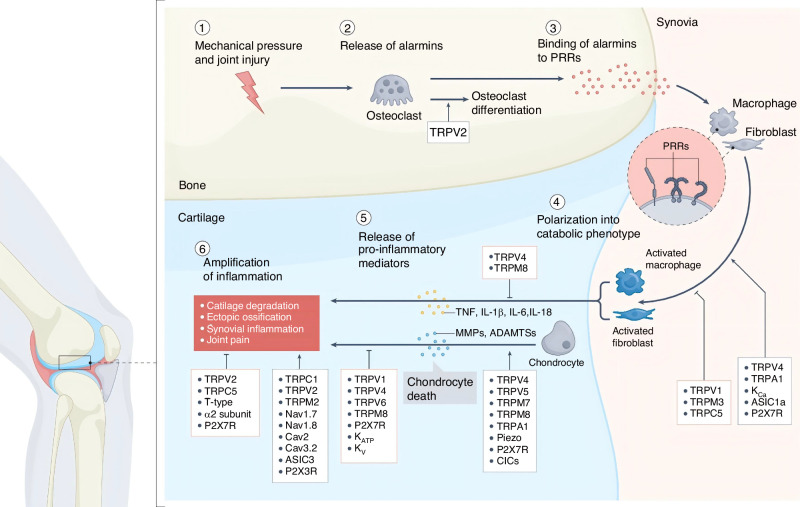
Ion channels play an important role in bone injury and repair. After the bone and joint injury, danger signals are triggered to alarm. These alarm signals bind to PRRs on immune cells, thereby promoting M1 macrophage polarization and fibroblast activation through ion channels, including TRPV4, TRPA1, and ASIC1a. The regulated release of pro-inflammatory cytokines (including TNF, IL-1β, IL-6, and IL-18) and matrix-degrading enzymes (such as matrix metalloproteinases, or MMPs, and a disintegrin and metalloproteinase, or ADAM) is controlled by ion channels (including Nav1.7, TRPV4, and Piezo) and amplifies the inflammatory response. This cascade reaction leads to cartilage degeneration, heterotopic ossification, synovial inflammation, and joint pain. PRR pattern recognition receptor, MMP matrix metalloproteinase, ADAM a disintegrin and metallo-proteinases.^129^ Copyright 2024, Springer Nature

During the differentiation process of BMSCs into osteoblasts, TRPV4 plays an important role. Specifically, through a mechanism related to fluid shear stress, it participates in regulation, together with Piezo1/2 and Ca^2+^-activated potassium (KCa) channels, among others, promoting the expression of genes related to osteoblast differentiation, such as Cox2 and Opn.^122^ It can also enhance collagen and calcium deposition through its chemical agonist GSK101.^135^ Additionally, it has been found that the membrane translocation of TRPM7 is enhanced by fluid shear stress. Intermittent fluid shear stress regulates the osteogenic differentiation of BMSCs through the TRPM7-Osterix axis, affecting the expression of Osterix and the activation of related signaling pathways such as p38 and Smad1/5.^136^ In addition, low-intensity pulsed ultrasound and nano-mechanical force generators can enhance the osteogenic effect of BMSCs by regulating TRPM7, the actin cytoskeleton, and intracellular calcium oscillations^137^ (Fig. 4).

The TRP family can also regulate the differentiation and functions of osteoclasts. For example, the calcium influx mediated by TRPV4 participates in the terminal differentiation of osteoclasts. Its activation can promote the formation of osteoclasts and affect the occurrence and development of osteoporosis. For example, in a mildly acidic environment, the activation of TRPV4 can encourage the formation of osteoclasts and is associated with their survival. Its deficiency will affect bone metabolism and increase the risk of osteoporotic fractures.^138^ TRPV1 participates in the differentiation process of osteoclasts. Its deletion will impair fracture healing and inhibit the differentiation of osteoclasts and osteoblasts. In an acidic environment, TRPV1 may be part of the acid-sensitive mechanism. Its antagonists can inhibit the formation and activity of osteoclasts, and have potential significance for the treatment of osteoporosis.^134^ Additionally, the TRP family influences calcium homeostasis in bones. It affects bone repair and remodeling by interfering with processes such as intestinal calcium absorption (TRPV6), renal calcium reabsorption (TRPV5), osteoclast differentiation (TRPV1, 2, 4, 5), and chondrocyte differentiation (TRPV4).^139^

In addition, other important types of ion channels are jointly involved in the occurrence, development, and bone diseases. Potassium channels are expressed in chondrocytes and respond to internal Ca^2+^. They have been shown to support ECM synthesis and chondrocyte proliferation.^140,141^ Acid-sensing ion channels (ASICs) are also expressed in chondrocytes, monocytes, and osteoblasts, and are associated with bone diseases (Fig. 4). ASIC triggers a rapid transient inward current upon acidification as a proton sensor, mediating the influx of Na^+^ and Ca^2+^.^142^ Chloride channels (ClCs) contribute to maintaining osmotic balance, cell volume, and cell potential homeostasis by facilitating the movement of chloride ions (Cl^−^) across the cell membrane.^143^ P2X receptors are ligand-gated cation channels that rapidly respond to extracellular ATP, thereby affecting physiological and pathological processes, including inflammation, pain perception, and neuromuscular transmission.^129,144^

In general, during bone regeneration, electrical signals regulate ion channel networks and interact with mechanical stimuli and the chemical microenvironment to drive the behavioral remodeling of BMSCs, macrophages, and vascular endothelial cells. Specifically, electrical stimulation activates CaV1.2, Piezo1 channels, and TRP channels, triggering intracellular calcium signal oscillations, which in turn regulate the osteogenic differentiation direction of BMSCs. Ca^2+^ activates the Runx2/OSX transcription program through the CaM-CaMK pathway, while also enhancing osteogenic phenotype expression through the Wnt/β-catenin and BMP/Smad signaling axes. For macrophages, electrical signals induce M2 polarization via Piezo1 channels, suppressing the NF-κB inflammatory pathway while promoting the secretion of anti-inflammatory factors such as TGF-β and IL-10, thereby creating an immunoregulatory chemotactic microenvironment. Endothelial cells perceive electro-mechanical signals through the Piezo1/TRPV4 channel, promoting VEGF-A secretion and endothelial cell migration. The activation of the PI3K/AKT pathway, together with intracellular calcium polarization, drives vascular network formation. Mechanical stress is converted into electrical signals through piezoelectric materials (such as BaTiO₃) or ECM reorganization (e.g., directed arrangement of actin filaments). The spatiotemporal release of chemical factors (e.g., BMP-2, SDF-1α) is regulated by membrane potential differences induced by electrical signals. This electro-mechanical-chemical coupling effect not only mimics the endogenous electrophysiological microenvironment of bone tissue but also precisely regulates cell fate determination and tissue reconstruction processes through multimodal signal synergy, providing a theoretical foundation for the development of intelligent bone repair materials.

## Electrically responsive hydrogels based on electric field stimulation

In bone tissue engineering, hydrogels have emerged as an ideal matrix for bone regeneration due to their high water content, three-dimensional porous structure, and biocompatibility. Their elastic modulus can be tuned across scales to match the requirements of different bone tissues, and they also exhibit injectability and self-healing properties. In recent years, researchers have developed electric field stimulation-responsive hydrogels by incorporating conductive polymers, piezoelectric materials, and other electrical materials. These hydrogels simulate the electrophysiological microenvironment of bone tissue or couple with biological signals, enhancing the efficiency of osteogenic differentiation. However, the design of such materials faces three core challenges: first, balancing matrix compatibility and mechanical properties—natural polymers have excellent biocompatibility but insufficient strength, while synthetic polymers offer controllable mechanical properties but lack cell adhesion sites; second, the regulation of structure morphology on function—the three-dimensional structure must match the porous hierarchical characteristics of bone tissue; third, the stable construction of conductive networks—the dispersion and interface compatibility of conductive fillers directly affect the efficiency of electrical signal conduction. Among these, integrating conductive functionality organically with the physiological requirements of bone tissue remains a key bottleneck constraining clinical translation. This chapter will systematically discuss three types of electric field-responsive hydrogel systems: conductive hydrogels, piezoelectric hydrogels, and other electric field-responsive systems. The focus will be on analyzing material selection, preparation methods, and related properties in material preparation.

### Conductive hydrogels

#### Design and functionality of conductive components

The matrix of conductive hydrogels is generally divided into natural or synthetic polymers. Natural polymers include alginate, chitosan, hyaluronic acid, and collagen. They possess excellent biocompatibility and biodegradability via enzymatic or hydrolytic reactions, as well as biological properties that promote wound healing. On the other hand, synthetic polymers, such as polyethylene glycol (PEG), poly(N-isopropyl acrylamide) (PNIPAAm), and polyvinyl alcohol (PVA), exhibit more controllable structures and superior mechanical properties; however, their poor cell adhesion, as well as their biocompatibility and degradability need to be considered.^145,146^ Natural polymers can be combined with synthetic polymers through simple mixing or cross-linking to integrate their advantages and obtain multiple functions, forming a variety of modified derivatives of natural polymers, such as GELMA, HAMA, and AlgMA.

However, the matrix materials used for fabricating hydrogels typically exhibit poor conductivity.^147^ Therefore, researchers usually add conductive materials to the hydrogel matrix to endow hydrogels with conductivity. Researchers commonly employ three methods: first, adding metal or metal salt ions, such as Ag, Cu, Na^+^, Li^+^, Ca^2+^, and Fe^3+^.^148^ For example, the PVA, N-(2-amino-2-oxoethyl)-2-acrylamide, and GelMA matrix itself is not conductive, but the final hydrogel exhibits ionic conductivity because NaCl is exogenously added, which ionizes into Na^+^ and Cl^-^, increasing the ionic concentration of the system, thereby achieving ionic conductivity.^149^ Adding metals and metal ions not only enhances the conductivity of the hydrogel but also confers additional functions, participates in the crosslinking of the hydrogel, and promotes its formation. For example, silver nanoparticles possess antibacterial properties and have been used in antibacterial dressings.^150^ Fe^3+^ can induce in situ pyrrolation and dopamine, giving the hydrogel strong wet adhesion performance.^151^ Under ultraviolet irradiation, Fe^3+^ is reduced to Fe^2+^, and in a low-temperature environment, Fe^2+^ catalyzes the decomposition of persulfate, generating free radicals and inducing monomer polymerization.^152,153^

**Fig. 5:**
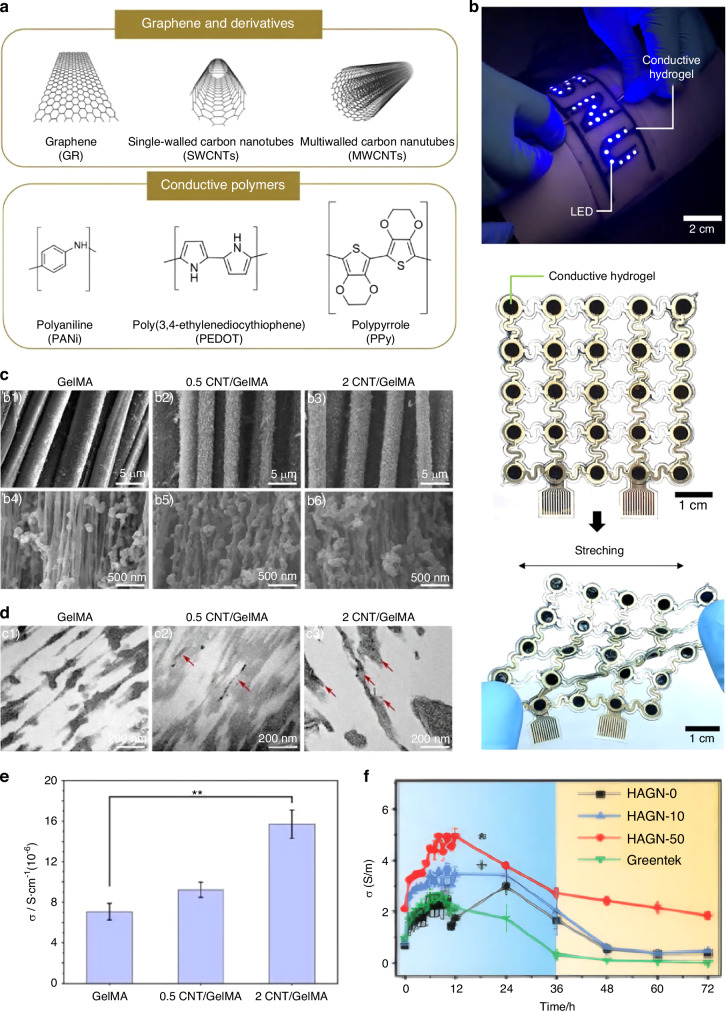
Microstructure and conductive properties of conductive hydrogels. **a** Microscopic molecular structures of graphene and conjugated π-polymers. **b** The conductive hydrogel exhibits superior conductivity and tensile properties, and can activate a light-emitting diode.^229^ Copyright 2024, American Association for the Advancement of Science. **c**, **d** SEM and TEM images of micron-sized fibers. **e** Conductivity of the conductive hydrogel.^230^ Copyright © 2024 Elsevier Ltd. **f** Conductivity of HAGN hydrogels containing different concentrations (0%, 10%, 50%) of graphite for 72 h.^231^ Copyright 2024, Wiley-VCH

Second, add polymer nanoparticles, such as carbon-based materials, conjugated π-polymers, and other polymer conductive materials (Fig. 5a). Carbon-based materials include carbon nanotubes (CNTs), graphene, and Mxene (a two-dimensional material with a graphene-like structure). Due to their excellent environmental stability, high conductivity, and low production cost, they are considered ideal materials for preparing conductive hydrogels.

The incorporation of CNTs and graphene nanosheets (GNPs) into PVA/CH/SA hydrogels enables the formation of a uniformly distributed three-dimensional conductive network composed of CNTs and GNPs. This network effectively transmits electrical stimuli and influences the behavior of osteoblasts cultured on the material surface.^147^ In addition to conductivity, CNTs and GNPs can typically enhance the mechanical properties and mechanical characteristics of hydrogels,^154,155^ thereby overcoming the application limitations of conductive hydrogels in electronic devices, high-load sensing, and other scenarios. For example, incorporating CNTs stabilized with LAPONITE® XLG nanosheets into hydrogels enables radical polymerization of 3-acrylamidobenzoic acid and acrylamide, resulting in non-covalent interactions within the hydrogel network. The hydrogel exhibits excellent mechanical properties, with tensile strength ranging from 252 to 323 kPa, a tensile strain of 880%–1 200%, a Young’s modulus of 48–50 kPa, and a fracture energy of 911–1 078 J/m². It also exhibits strong elasticity and fatigue resistance after 1 000 consecutive tensile and compressive cycles.^156^

Conjugated π-polymers are based on benzene rings, such as polyacetylene, poly[3,4-(methylenedioxy)thiophene] (PEDOT), polypyrrole (PPy), and polyaniline. This structural feature enables electrons to move freely within the unsaturated skeleton, thereby achieving conductivity.^157,158^ PPy is a conductive polymer with excellent electrical conductivity, ease of synthesis, and stability, commonly used as the conductive component in conductive hydrogels. Fan Wang et al. innovatively combined the conjugated π-polymer PPy with piezoelectric materials to construct a stress-electric coupling hydrogel microsphere. By leveraging the characteristic of free electron movement in conjugated π-polymers, they enhanced the internal electron transfer efficiency of the microspheres, thereby reducing losses in stress-to-electricity conversion. This discovery offers a strategy for low-loss transduction between tissues, particularly those with mechanically sensitive tissues.^159^ Additionally, it enhances excellent photothermal conversion capability. In GOMP hydrogels, under 808-nanometer laser irradiation, the photothermal conversion efficiency reaches 31.61%.^160^ PEDOT doped with polystyrene sulfonate (PSS), known as PEDOT:PSS, is one of the most commonly used conductive polymers today. The hydrophilic polyanion PSS acts as a dopant while enabling the polymer to be processed from an aqueous solution. Due to its mixed electronic and ionic conductivity, ease of processing, excellent water and air stability, and biocompatibility, PEDOT:PSS has been widely utilized in wearable electronics and bioelectronics.^161,162^

In addition, semiconductors, which have conductivity between conductors and insulators, are often incorporated into hydrogels to enhance their conductivity. Black phosphorus (BP) nanosheets, a type of semiconductor, exhibit good biocompatibility and a thickness-dependent bandgap, ranging from 0.3 eV in bulk to 2.0 eV in a single layer, allowing them to absorb a wide range of light in the visible light region.^163^ This structural characteristic enables BP nanosheets to be widely incorporated into hydrogels, enhancing the conductive and photosensitive properties of the materials. For example, Kun Liu’s team incorporated tannic acid (TA)-modified BP nanosheets (BP@TA) into a chitosan hydrogel matrix (LAMC) to improve its conductivity. In a rat model of complete spinal cord injury, implanting the LAMC/BP@TA hydrogel enhanced the recovery of motor function in rats with complete spinal cord injury.^164^ Chao Xu’s team incorporated polydopamine-modified silicon-phosphorus (SiP@PDA) nanosheets into a mixture of gelatin methacryloyl and decellularized extracellular matrix (GelMA/ECM) to construct a biodegradable, conductive hydrogel, thereby promoting nerve tissue repair.^165^ Using the free radical polymerization method, Chenyu Sang’s team prepared a conductive hydrogel (BPTP) with excellent mechanical, anti-fatigue, and photo-responsive properties. This hydrogel is composed of polyacrylamide as the matrix, 2,2,6,6-tetramethylpiperidine-1-oxyl oxidized cellulose nanofibers as the reinforcing agent, and polydopamine-modified black phosphorus (BP@PDA) as the photosensitizer. The sensor based on BPTP exhibits high cycling stability, excellent strain sensitivity (GF = 6.0), and pressure sensing ability (S = 0.13 kPa⁻¹), making it suitable for monitoring human activities.^166^

#### Selection of crosslinking methods

The cross-linking of hydrogels refers to the process of forming a three-dimensional network structure through chemical bonds or physical interactions. With its three-dimensional network and porous structure, hydrogels can absorb a large amount of exudate, maintain a moist environment, and serve as carriers for bioactive substances and cells.^167^ However, they may lack a firm supporting structure, and the water is prone to evaporation, resulting in the inability to be used for a long time.^168^ These problems can be addressed by influencing the structure of hydrogels through different crosslinking methods. According to different action mechanisms, crosslinking methods can be divided into covalent bond crosslinking and non-covalent bond crosslinking. Covalent bond crosslinking encompasses amide bonds, Schiff base bonds, acyl hydrazone bonds, boronate esters, and other similar bonds. Non-covalent bonds include ionic bonds, hydrogen bonds, electrostatic interactions, host-guest interactions, and hydrophobic interactions.^169^ Generally speaking, permanent covalent bond crosslinking usually results in a rigid matrix. In contrast, physical crosslinking and dynamic covalent bonds typically result in a soft structure.^170^

The addition of conductive components will affect the choice of crosslinking methods. When preparing conductive hydrogels with carbon-based materials, such as CNTs or graphene, as conductive additives, unique methods can be employed to combine and crosslink the conductive components with the polymer matrix, thereby achieving excellent conductivity and dispersion. For example, it may be necessary to perform surface modification on the conductive elements to enhance their compatibility and interaction with the polymer. When Seifi et al. added CNTs and GNPs to the synthesized PVA/CH/SA hydrogel, they carboxylated the CNTs in advance to enhance their dispersion in the solution and improve biocompatibility. Multiple crosslinking methods were adopted. Sodium alginate underwent ionic crosslinking under the action of calcium ions, and the physical crosslinking of PVA was achieved by repeating the freeze-thaw process three times.^147^ For conductive hydrogels with ionic liquids as conductive components, ionic liquids can alter the solution properties and reactivity of polymers, thereby affecting the crosslinking methods. Meanwhile, the ions in ionic liquids may also participate in the crosslinking reaction, forming ionic bond crosslinking or influencing the formation of other chemical bonds, which differs from ordinary hydrogels that rely solely on the functional groups of polymers for crosslinking.^171^ In the novel double-network ionic conductive hydrogel (PMP-DN-ICH) based on the poly(ionic liquid)/Mxene/polyvinyl alcohol system synthesized by Zhao’s team, there were many ionic liquid monomers. During the crosslinking process, the physical crosslinking network of PVA was first formed through freeze-thawing. Then, in-situ polymerization/crosslinking reactions were initiated using ionizing radiation technology to form the ionic liquid monomer VBImBr and the crosslinking agent Ph-3MVIm-Br, which polymerized within the PVA network to create a chemically crosslinked poly (ionic liquid)- poly (vinyl alcohol) (PIL-PVA) network. Subsequently, the added Mxene nanosheets could form non-covalent interactions (such as hydrogen bonds) with PVA and PIL, further strengthening the mechanical properties of the hydrogel.^172^

The cross-linking of conductive hydrogels will also directly affect their conductive properties and pore sizes. Some crosslinking methods may limit the transport of ions or electrons, thus reducing the conductive properties. For example, excessive chemical crosslinking may lead to an overly dense polymer network, hindering the movement of conductive ions or electrons.^173^ Therefore, when preparing conductive hydrogels, it is necessary to choose appropriate crosslinking methods to balance the mechanical and conductive properties. For example, Zhang’s team prepared a double-network hydrogel by combining physical and chemical crosslinking. The chemically cross-linked network, composed of polyacrylamide, ensured the mechanical strength of the hydrogel. The complexation of divalent calcium ions with the carboxyl groups in sodium alginate formed a physical crosslinking network, endowing the hydrogel with excellent resilience and high sensitivity.^174^ Additionally, the superposition of multiple crosslinking methods can affect the pore size of the hydrogel. In the GelMA-PPy-Fe hydrogel, the addition of PPy and Fe^3+^ gradually reduced the pores of the hydrogel from the original (32.27 ± 1.65) μm (GelMA) to (27.56 ± 1.81) μm (GelMA-PPy) and then to (11.23 ± 1.05) μm (GelMA-PPy-Fe).^175^ It has been confirmed in in vivo experiments that a higher porosity and pore size will lead to better bone ingrowth. While in vitro, low porosity stimulates osteogenesis by inhibiting cell proliferation, forcing cell aggregation, and adsorbing bone-indicating proteins and ion exchange. However, a lower pore size and porosity often mean the hydrogel has better mechanical strength^176^ (Fig. 5C–E, Fig. S1B). Therefore, selecting suitable materials, employing suitable crosslinking methods, and designing more selective ones are necessary to meet the specificity required for bone repair.

#### Physicochemical properties and their impact on bone regeneration

With the addition of conductive materials, the types of properties that need to be considered for hydrogels increase accordingly. Some metals and carbon nanomaterials are insoluble in water and tend to aggregate or disperse, which decreases their electrical conductivity. Therefore, the uniform dispersion of conductive substances is of great importance. Researchers often use techniques such as scanning electron microscopy (SEM) to observe the surface and internal microscopic structures of hydrogels, including pore size and pore distribution, to ensure the uniform distribution of conductive materials.

More importantly, some conductive materials or the substances produced during their degradation in vivo may have toxic effects on cells, trigger immune responses, and change the local microenvironment, which is not conducive to maintaining the normal physiological functions of cells and tissues. After ES treatment, the degradation rate of hydrogels may accelerate, and the release rates of organic and inorganic components in hydrogels during degradation will also increase. For example, within 24 days after applying electrical signals to the CPM@MA hydrogel, the mass loss increased from (23.82 ± 2.87)% to (55.35 ± 4.21)%. Compared with that in simulated body fluid, the release rates of CaP and MgTiO₃ increased by 1.12 times and 0.89 times, respectively, after the application of ES.^175^ Therefore, evaluating the biocompatibility of materials is a step that cannot be bypassed. Frequently performed in vitro experiments include cytotoxicity testing and microscopic observation of cell morphology.^177^ In vivo experiments include acute toxicity tests and long-term toxicity tests. The acute toxicity test aims to observe changes in body weight and gross organ morphology of experimental animals over a short period of time. The long-term toxicity test assesses long-term toxicity by implanting bioactive materials for an extended period and evaluating blood biochemical indexes and histological staining. In addition to this, drug release profiles are usually required for drug-loaded hydrogels. For hydrogels with medium- to long-term applications, material degradation rates, degradation patterns, and the removal of degradation products need to be evaluated through in vivo and in vitro experiments. For example, Cheng et al. recorded the degradation ability of hydrogels in response to chelating agents by observing the morphological changes of the hydrogels in real time; a control group with different concentrations of chelating agents was set up to ensure the controllability of the degradation time. After the hydrogels were implanted in mice with breast cancer, the residual amount and degree of fragmentation of the hydrogels were assessed over time. For the degraded products, whether DEPs could be safely removed was tested by analyzing the in vivo distribution of platinum nanoparticles.^178^

Biomaterials for bone tissue engineering typically require a specific mechanical strength to support the process of bone tissue regeneration and also need to exhibit a certain degree of mechanical deformation ability to adapt to the movement postures of the human body. Therefore, measuring the mechanical characteristics of conductive hydrogels, including elastic, viscoelastic, and mechanical strength properties, as well as fluid mechanics-related characteristics, is necessary. By using compression tests to obtain the compressive stress-strain curves of hydrogels and then calculating parameters such as Young’s modulus, the ability of hydrogels to deform and resist damage when subjected to external forces can be macroscopically evaluated.^179–181^ An atomic force microscope was used to measure the elastic modulus of hydrogels, operating in tapping mode in air and calculating Young’s modulus using the Hertz model. This method can obtain the microscopic local elastic mechanical properties.^180,182^ Using a rotational rheometer to measure the rheological properties of hydrogels, including storage modulus (G′), loss modulus (G″), and elastic modulus. The GelMA-PPy-Fe hydrogel synthesized by Huang’s team exhibited typical solid behavior (G′ > G″) at low temperatures, with an elastic modulus higher than that of the GelMA hydrogel. The tensile strength of the GelMA-PPy-Fe hydrogel was 0.028 MPa (28 kPa), Young’s modulus was 0.16 kPa, the compressive strength was approximately 3.8 MPa, and the toughness was approximately 324 kJ/m^3^. The compressive strength was similar to that of human cancellous/trabecular bone (0.1–16 MPa).^153^

Electrical conductivity is a key indicator for measuring the conductive ability of hydrogels. Generally speaking, the more conductive materials are added, the higher the electrical conductivity and the better the conductive performance will be (Fig. 5f and Fig. S1C). The higher the signal transmission efficiency, the better it will be in applications such as electronic devices and biosensors. By observing the changes in parameters such as electrical conductivity, resistance, and electrode potential under different conditions of voltage, current density, and electrolyte concentration, the electrochemical stability of hydrogels can be known, which is particularly important in some applications that require repeated charging and discharging or long-term stable electrical signal transmission, such as flexible batteries and wearable electronic devices (Fig. 5b and Fig. S1A). For example, G M Shanthini et al. used impedance analysis to test the conductivity of the hydrogel. The Nyquist plot shows that the resistance decreases with an increase in ion energy, and the conductivity values of the samples implanted with 1e15, 5e15, and 1e16 ions are 1.59 μS/cm, 1.91 μS/cm, and 2 μS/cm, respectively.^179^ In addition, according to the application requirements of conductive hydrogels, the different characteristics of hydrogels can be tested in a targeted manner, such as antibacterial ability^147,181^ and ion release ability.^153,180^ Table 1 summarizes the key electrical properties and osteogenic performance of conductive hydrogels, including matrix compositions and crosslinking strategies. The data provide a comparative overview of material designs for bone regeneration applications.

**Table 1:** Electrical characteristics of conductive hydrogels in promoting bone healing

Matrix	Conducting materials	Crosslink	Micro-structure	Diameter	Conductivity	Ref.
MA	PEDOT:PSS, MgtiO3	noncovalent bond	granular	-	C@MA:0.44 ± 0.03 mS/cm CP@MA:1.29 ± 0.08 mS/cm CPM@MA:1.52 ± 0.09 mS/cm	^175^
GelMA	PPy, Fe^3+^	noncovalent bond, covalent bond	porous meshwork	(11.23 ± 1.05) μm (GelMA-PPy-Fe)	GelMA-PPy-Fe:9.6 ± 1.3 mS/cm	^153^
GelMA	Mg^2+^, BP nanosheets	covalent bond	granular	250–300 μm	0.10 S/m	^180^
GelMA	BP nanosheets	covalent bond	granular	*d* = 200 μm	0.22 S/m	^213^
GelMA	PPyNWs	covalent bond	porous meshwork	8 ± 4.1 μm	0.263 ±0.020 S/m (GelMA@4PPy)	^214^
Carboxymethyl chitosan	CNT	covalent bond	porous meshwork	50–80 μm	-	^181^
PVA, CH, SA	MWCNT-COOH, GNP	noncovalent bond	porous meshwork	12.1 ± 26.6 μm(PVA/CH/SA) 13.9 ± 30.0 μm(+CNT) 17.0 ± 35.3 μm (+CNT + GNP)	(1.8 ± 0.02) × 10^−5^ S/m(PVA/CH/SA) (5.7 ± 0.24) × 10^−2^ S/m(PVA/CH/SA-CNT) (6.3 ± 0.05) × 10^−3^ S/m(PVA/CH/SA-GNP)	^147^
PVA	N^3+^ ion	noncovalent bond	platy	-	1.59 μS/cm (1e15) 1.91 μS/cm (5e15) 2 μS/cm (1e16)	^179^
Acrylamide, gelatin, deacetylated chitosan	Mo2Ti2C3, MXene	covalent bond	porous meshwork	-	50 mS/cm (20 μg/mL MXene) 55 mS/cm (40 μg/mL MXene) 60 mS/cm (80 μg/mL MXene)	^235^
PAA	GO	covalent bond	porous meshwork	2–10	17 × 10^−4^ S/cm (0.2% GO-PAA) 25 × 10^−4^ S/cm (0.4% GO-PAA) 33 × 10^−4^ S/cm (0.6% GO-PAA)	^236^
Gel	PEDOT: PSS	covalent bond	porous meshwork	50–250 μm (0.3% PEDOT:PSS) 70–150 μm (0.1% PEDOT:PSS)	170 μS/m (0.3% PEDOT:PSS) 100 μS/m (0.1% PEDOT:PSS)	^237^
GelMA	MWCNT	covalent bond	spherical pellet	-	9.21 S/cm (70-30-5)	^238,239^
OPF gel	CNT-PEG-acrylate (CNT-pega), BP	covalent bond	tubular-like morphological features	-	0.008 S/m (CNTpega 16 mg/mL, BP 0.8 mg/mL)	
GP, OP	PPy	covalent bond	porous meshwork	173.13 μm (G-OP) 295.96 μm (GP15-OP)	0.06 mS/m (G-OP) 2.14 ± mS/m (GP15-OP)	^240^
RSF	MXene	noncovalent bond, covalent bond	porous meshwork	-	4 × 10⁻⁴ S/cm (8 mg/mL MXene)	^187^
ECM, AlgMA	BP@PDA	covalent bond	porous meshwork	-	0.059 S/m	^211^

*MA* methacrylated alginate, *GelMA* gelatin methacryloyl, *BP* black phosphorus, *PVA* polyvinyl alcohol, *CH* chitosan, *SA* sodium alginate, *PAA* polyacrylic acid, *GO* Graphene oxide, *Gel* gelatin, *OPF* oligo(poly(ethylene glycol) fumarate, *CNT* carbon nanotubes, *PEG* poly(ethylene glycol), *RSF* regenerated silk fibroin, *CALM* calmodulin, *GP* gelatin-graft-polypyrrole, *OP* oxidized pectin, *AlgMA* alginate methacryloyl

### Piezoelectric effect hydrogels

#### Design and functionality of piezoelectric components

Unlike conductive hydrogels, some piezoelectric hydrogels can utilize piezoelectric materials directly as the hydrogel matrix. According to different material sources, they can be divided into natural polymer-based and synthetic polymer-based ones. One of the most common natural biopolymers is silk fibroin (SF), which can be easily extracted from silkworms, spiders, or other natural sources and has excellent biocompatibility and biodegradability.^183^ Due to its internal structure, which usually having highly crystalline β-sheets formed among hydrophobic regions by interactions such as hydrogen bonds, van der Waals forces, and hydrophobic interactions, SF has mechanical robustness and endows SF with inherent piezoelectricity (d14 ≈ −1.5 pC/N) according to its β-sheet crystallinity and crystal orientation.^184,185^ Some studies have employed various methods to enhance the piezoelectric effect of SF and endow it with more diverse functions. For example, Senthil Kumar Rathinasamy’s team combined SF with carbon nanofibers to prepare a piezoelectric sensor.^186^ Hu’s team combined SF with Mxene to endow the hydrogel with conductivity, promote bone regeneration, and simultaneously utilize it as a piezoresistive pressure sensor to monitor the electrophysiological microenvironment.^187^ Chen’s team prepared a 3D-printed piezoelectric catalytic SF-MA/PEGDA/Ag@BT hydrogel with excellent piezoelectric catalytic performance, which can induce piezoelectric polarization to generate reactive oxygen species under ultrasonic conditions, thereby enhancing antibacterial activity and the healing process of infected wounds.^188^ A representative synthetic polymer is PVDF, along with its copolymers. PVDF has five possible crystalline forms (α, β, γ, δ, and ε). Among them, the β-crystalline form and its copolymer poly(vinylidene fluoride-co-trifluoroethylene) (PVDF-TrFE) have strong piezoelectric properties (−32 and −28 pC/N).^158,189,190^ This material can be induced to form more β-crystalline forms through physical methods, such as stretching and annealing, or chemical processes, such as adding nucleating agents, thereby endowing the hydrogel matrix itself with piezoelectric properties. PVDF is primarily used as a medical suture due to its long-term mechanical and chemical stability in a mild hydrolytic environment in vivo.^191^

**Fig. 6:**
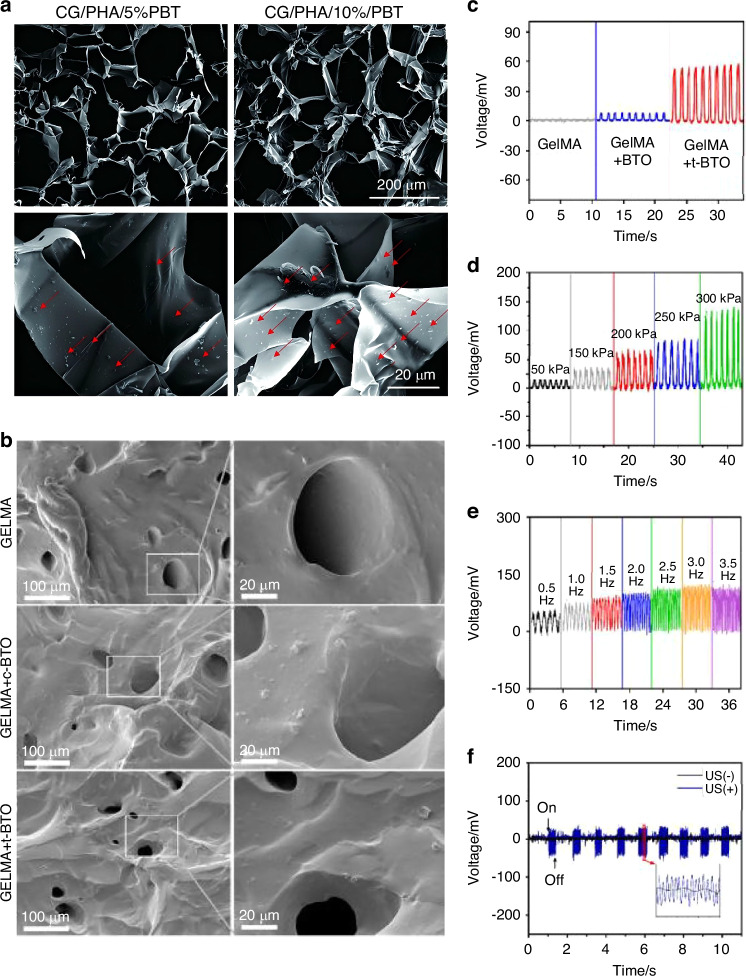
Structures and piezoelectric capabilities of piezoelectric hydrogels. **a** Dopamine-modified barium titanate participates in the crosslinking of hydrogels. Representative SEM images of different hydrogel samples, where the red arrows indicate piezoelectric nanoparticles.^194^ Copyright 2023, Springer Nature. **b** SEM images of GelMA, GelMA + c-BTO and GelMA + t-BTO. **c**–**f** Electromechanical responses of various hydrogels before and after ultrasonic stimulation under 200 kPa, 50–300 kPa, 0.5–3.5 Hz.^220^ Copyright © 2024 Elsevier Ltd

Piezoelectric hydrogels can also utilize non-piezoelectric materials as the matrix and acquire piezoelectric characteristics by incorporating piezoelectric components. BTO and poly-L-lactic acid (PLA) are the most common piezoelectric components.^192^ Barium titanate is an inorganic piezoelectric material with high piezoelectricity (d33 = 190 pC/N), good biocompatibility, and ease of synthesis.^193^ Typically, BTO nanoparticles are challenging to uniformly disperse in the hydrogel matrix due to their considerable weight and limited interaction with polymer chains. To solve this problem, specific interactions between BTO nanoparticles and polymer chains are required. Both amino-modified barium titanate and polydopamine-modified barium titanate are promising ideas (Fig. 6a and Fig. S1D) that can uniformly distribute barium titanate nanoparticles within the hydrogel, enhance the piezoelectric performance, and improve the storage modulus (532.2 Pa) of the hydrogel.^194,195^ However, barium titanate’s poor biodegradability and low doping level limit its applicability for in vivo implantation.^193,196^ PLA is a natural polymer with four crystal conformations, among which the β form is piezoelectric (10 pC/N).^197^ As early as 1996, Y. Ikada et al. discovered the piezoelectric characteristics of PLA. By intramedullary implanting PLLA rods into the incisions of cat tibias for fixation for 8 weeks and observing the formation of callus, it was proved that the promotion of fracture healing by stretched PLLA fixation could be attributed to the piezoelectric current generated by strain during leg movements.^198^ PLA has been used as bone implants and soft tissue fillers in the United States and is considered biocompatible and safe.^199,200^ However, PLA has a low degradation rate (1–5 years), and the acidic metabolites produced by its decomposition can cause inflammatory reactions at the implantation site, which may lead to delayed and adverse inflammatory reactions.^200,201^ Therefore, safer and more effective piezoelectric components need to be discovered. In recent years, Liu’s team self-assembled phenylalanine into diphenylalanine (FF) and proposed a piezoelectric conductive scaffold composed of piezoelectric cartilage-decellularized extracellular matrix (dECM) and piezoelectric conductive modified gelatin (Gel-PC). This scaffold forms a hexagonal structure with strong piezoelectric properties, with a d33 value of 18 pm/V. Its piezoelectric performance meets the ES intensity requirements of cartilage (25 mV) and bone (40–80 mV), exhibiting superior biocompatibility and degradability, and has great potential in clinical applications^202^

#### Integration and crosslinking strategies

For piezoelectric hydrogels with piezoelectric materials directly serving as the hydrogel matrix, a 3D network can be formed through multiple crosslinking methods. For example, SF hydrogels can form hydrophobic interactions and hydrogen bonds through various methods such as enzyme-mediated dityrosine crosslinking,^187^ β-sheet physical crosslinking,^203^ Fenton reaction-mediated dityrosine crosslinking,^204,205^ photo-initiated dityrosine crosslinking,^206^ and methacrylate-modified photo-crosslinking.^207^ The impact of different crosslinking methods on its piezoelectric effect is not yet evident. Several studies have demonstrated that modifying piezoelectric elements can influence the crosslinking strength of hydrogels. For example, by modifying barium titanate with amino groups, the crosslinking strength between KBTO nanoparticles and the organic network of chondroitin sulfate/gelatin is enhanced, which in turn improves the mechanical properties of the prepared hydrogel and is beneficial in strengthening the adhesion effect of the hydrogel on bone tissue.^195^ (Fig. S2) However, in most cases, piezoelectric elements do not directly participate in the cross-linking of hydrogels.^192^ Therefore, for hydrogels with additional piezoelectric elements, it is crucial to uniformly integrate these elements into the 3D network of the hydrogel.

#### Physicochemical Properties and Their Impact on Bone Regeneration

Like conductive hydrogels, the addition of piezoelectric elements may generate toxic substances in cells, trigger immune responses, alter the local microenvironment, and is not conducive to maintaining the normal physiological functions of cells and tissues. If they are difficult to degrade in vivo, they will likely cause delayed healing or non-union of bone injuries. Therefore, it is necessary to test the biocompatibility and degradability of piezoelectric hydrogels. Similarly, piezoelectric hydrogels are often made into wearable devices for the human body. Compared to conductive hydrogels, they need to adapt to the movement postures of the human body more frequently and require a specific deformation ability. Therefore, it is crucial to evaluate the mechanical properties of piezoelectric hydrogels. To ensure the uniform distribution of piezoelectric elements, researchers apply various techniques for detection. Fourier transform infrared spectroscopy (FTIR), Zeta potential, SEM, and confocal laser scanning microscopy are commonly used detection methods. Vinikoor’s team used rhodamine B dye mixed into the PLLA film to verify the uniform distribution of NF-sPLLA within the collagen hydrogel.^208^

Piezoelectric performance is a crucial indicator for evaluating the functionality of piezoelectric hydrogels. Among them, the piezoelectric constant d33 is used to quantify the piezoelectric performance, expressed as C/N (coulombs per newton) or m/N (meters per newton). It reflects the material’s ability to generate charges when subjected to a force or to deform under the action of an electric field. The higher the value, the stronger the piezoelectric performance of the material. Professional testing equipment, such as a quasi-static d33 meter, is usually used for accurate measurement. Besides d33, the piezoelectric performance of piezoelectric hydrogels can also be evaluated by measuring the voltage or current output generated when subjected to external pressure or vibration. The magnitude and stability of these electrical signals are directly related to the performance of piezoelectric hydrogels in practical applications. Higher piezoelectric performance means that piezoelectric hydrogels can generate stronger electrical signals under the same external stimulation and thus better achieve their functions^209^(Fig. 6b–f).

Additionally, according to the application requirements of piezoelectric hydrogels, the characteristics of different hydrogels, such as antibacterial properties, can be tested and targeted. Table 2 outlines the piezoelectric characteristics and degradation profiles of piezoelectric hydrogels, emphasizing the influence of matrix materials and piezoelectric additives. These data illustrate the relationship between material structure and bone-regenerative efficacy.

**Table 2:** Electrical characteristics of piezoelectric hydrogels in promoting bone regeneration

Matrix	Piezoelectrical materials	Crosslink	Output voltage	Degradation	Elastic modulus	Ref.
dECM, Gel-PC	FF	noncovalent bond	20 mV (dECM-P)	1 000 cycle, 21 d	-	^202^
Cs/Gel	PHA, PBT	covalent bond, noncovalent bond	0.8 V (G/PHA/10%PBT)	-	342.8 Pa (CG) 408.4 Pa (CG/PHA) 493.1 Pa (CG/PHA/5%PBT) 532.2 Pa(CG/PHA/10%PBT)	^194^
collagen	PLLA, NF-sPLLA	noncovalent bond	33.7 mV (NF-sPLLA)	-	-	^208^
OCS, Gel	BTO, KBTO	covalent bond, noncovalent bond	41.16–61.82 mV (1.5 W/cm^2^) 45.19–65.68 mV (2 W/cm^2^)	-	2 160.00 ± 73.94 Pa (GO) 2 407.50 ± 115.00 Pa (0.1KBGO) 2 560.00 ± 129.87 Pa (0.5KBGO)	^195^
GelMA	BTO	covalent bond	10 mV/mm^2^ (3 N, 2 Hz)	40%(14 d)	-	^241^
GGMA	CoFe_2_O_4_/PVDF	noncovalent bond	d33 = 22 pC/N	-	20.33 kPa (0.5 wt%) 26.70 kPa (1 wt%) 28.69 kPa (2 wt%)	^242^
CS/HAp, GM/Alg	PWH nanoparticles	covalent bond, noncovalent bond	d33 = 9.41 pC/N	46%(35 d)	1.02 MPa (Bio-S)	^243^

*dECM* decellularized extracellular matrix, *Gel-PC* piezoelectric-conductive modified gelatin, *FF* diphenylalanine, *PLLA* poly-L-lactic acid, *NF-sPLLA* nanofibers of PLLA, *Cs/Gel* chitosan/gelatin, *PDA* polydopamine, *PHA* PDA-modified ceramic hydroxyapatite, *PBT* PDA-modified barium titanate, *OCS* oxidized chondroitin sulfate, *BTO* barium titanate, *KBTO* amino-functionalized BTO, *GGMA* methacrylate gellan gum, *PDVF* poly(vinylidene fluoride), *CS/HAp* chitosan-hydroxyapatite, *GM/Alg* calcium-chelated biocompatible alginate and covalently crosslinked gelatin methacryloyl, *PWH* piezoelectric whitlockite

### Other hydrogels responsive to electrical stimulation

**Fig. 7:**
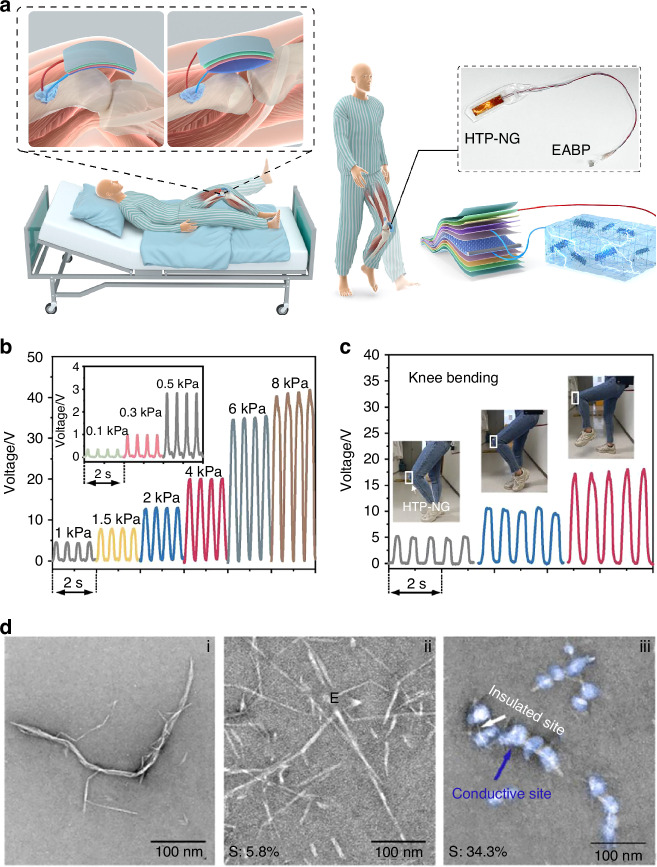
Other electrical hydrogels (**a**–**c**) Nanotriboelectric generator combined with conductive hydrogel. **a** A schematic diagram of a fully implantable battery-free BD-ES system allows patients to perform active or passive functional exercises under guidance. **b**, **c** Output voltage signals of HTP-NG under different pressures and in response to knee bending.^211^ Copyright 2024, American Association for the Advancement of Science. **d** The combination of the drug and the hydrogel under electrostatic adsorption. Transmission electron microscope (TEM) image.^232^ Copyright 2024, American Chemical Society

When materials based on the triboelectric effect come into contact with different materials and undergo relative motion due to the triboelectric effect, charge transfer occurs on the surface of the hydrogel, thereby forming a potential difference and generating an electrical signal. Triboelectric nanogenerators are the main application based on triboelectric materials. This is a distributed energy harvesting device based on the coupling effect of contact electrification and electrostatic induction.^210^ In research on promoting bone repair, Tianlong Wang’s team developed a nanogenerator that combines piezoelectric and triboelectric effects. Its structure consists of a PVDF film (piezoelectric layer) and a polytetrafluoroethylene (PTFE) and silver (Ag) electrode layer (triboelectric layer). When an external force is applied to the hybrid tribo/piezoelectric nanogenerator (HTP-NG), for example, during knee joint movement, the PVDF film bends, generating a piezoelectric potential. At the same time, charge transfer occurs between the bent Ag layer and the PTFE layer due to the triboelectric effect, thus generating a potential difference. The synergistic effect of triboelectricity and piezoelectricity enables the HTP-NG to convert mechanical energy into electrical energy, generating biphasic electrical pulses that serve as a form of biofeedback. When used in the BD-ES system, it can promote osteogenesis-related biological processes, including calcium ion influx and osteogenic differentiation. In a rat femoral defect model, through BD-ES and subsequent bone mineralization treatment, the bone defect was reversed, promoting bone tissue regeneration^211^(Fig. 7a–c).

In addition, utilizing electrostatic adsorption to enhance the interaction between drugs and hydrogels has also been a research focus in recent years (Fig. 7d and Fig. S1E, F). Tao et al. utilized the positive charge of quaternized chitosan and the negative charge of carboxylated chitosan to self-assemble into nanoparticles through electrostatic adsorption during the preparation of vancomycin nanoparticles (VCM-NPs). The VCM-NPs formed through electrostatic interaction were embedded into CS-Gel (chitosan temperature-sensitive hydrogel) to construct an injectable temperature-sensitive VCM-NPs/Gel drug delivery system. This system enhances the encapsulation efficiency and drug loading of VCM-NPs, facilitating the sustained release of vancomycin, which aids in local anti-infection treatment and provides a favorable environment for bone regeneration.^19^ Lin et al. When preparing a composite hydrogel to promote the healing of osteomyelitis, since the hyaluronic acid aqueous solution is negatively charged and the adopted antibiotic solution is positively charged, the antibiotics were combined into the composite hydrogel through electrostatic interaction. This enables the composite hydrogel to continuously release antibiotics over an extended period, playing a key role in sterilization and preventing wound infections. Moreover, by loading BMPs into the composite hydrogel through slow-release microspheres, the proteins can be continuously released over an extended period to induce new bone formation and promote bone healing.^212^

## Electric field stimulation-responsive hydrogels promote bone regeneration

### Providing electrical signals to promote cell activity

Simulating the electrophysiological characteristics of the natural bone matrix is a key feature of responsive hydrogels that are stimulated by an electric field. By adding electrical materials and adjusting the components, structures, and proportions, exogenous ES can be applied to simulate the electric field characteristics within bones. This promotes cell differentiation, proliferation, and migration, providing bone cells with a growth environment similar to that in vivo, and creates favorable environmental conditions for regeneration.^179^ Among them, conductive hydrogels can generate electrical signals in vivo or in vitro, simulating the electrophysiological environment of natural bone tissues. Adding piezoelectric materials enables hydrogels to generate electricity spontaneously, simulating the electric field generated by bones during human activities.^202^ Conductive materials and piezoelectric materials are not mutually exclusive.

**Fig. 8:**
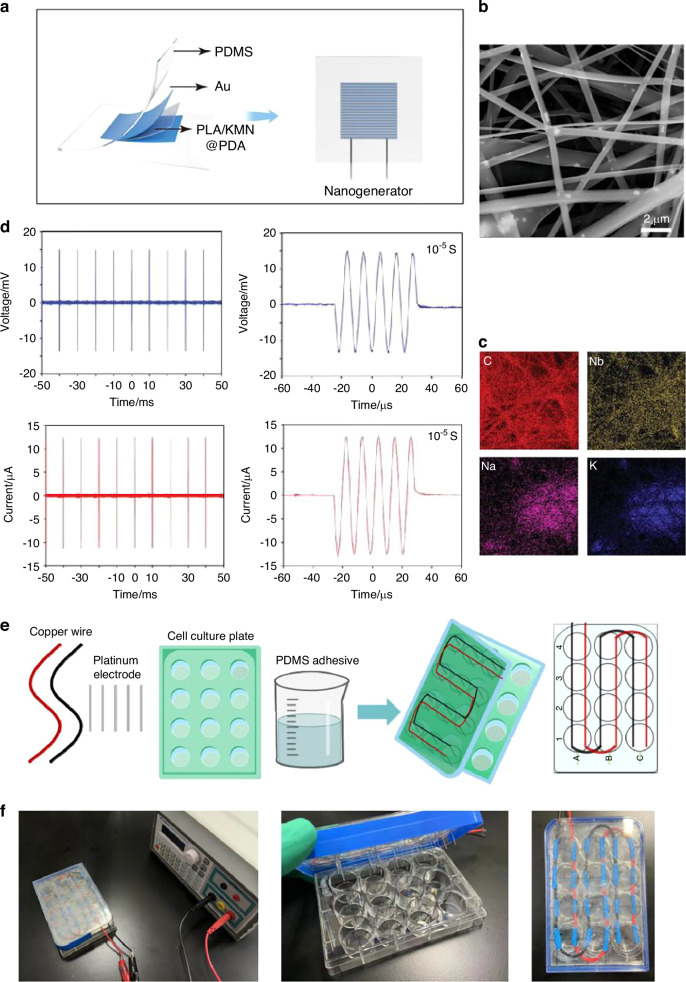
Conductive devices **a**–**d** Characterization of the PLA/KNN@PDA thin-film nanogenerator. **a** Schematic illustration of the structure of the PLA/KNN@PDA nanogenerator. **b** SEM image of the PLA/KNN@PDA nanofibers (scale bar: 2 µm). **c** EDS elemental mapping images of the PLA/KNN@PDA nanofibers. **d** Open-circuit voltage and short-circuit current generated by the PLA/KNN@PDA thin-film nanogenerator under ultrasonic stimulation.^213^ Copyright 2024, Wiley-VCH. **e**, **f** Schematic diagrams and photographs of the fabrication process of the self-made ES device.^214^ Copyright 2024, American Chemical Society

Diverse requirements have led to the introduction of various materials into the hydrogel system. Some studies introduce conductive and piezoelectric materials simultaneously to form composite polymer materials. The intensity of these electrical stimuli can be adjusted according to the proportion of materials introduced. There are various ways to apply ES. In some studies, self-contained power generation devices are often designed to utilize the electrical activity of electrical hydrogels better. For example, Jiwei Sun’s team integrated a wireless piezoelectric nanogenerator during the fabrication of conductive hydrogel microspheres loaded with DPSCs. They prepared a piezoelectric film using a mixture of the polymer PLA and K0.5Na0.5NbO3 (KNN, piezoelectric dopant) nanowires, which could be programmed by modulating ultrasonic pulses. Further tests showed that the output voltage was 15 V and the output current was 12.6 µA^213^(Fig. 8a–d). Xia’s team initially designed to apply current using cell culture plates to achieve efficient ES in the experimental group. Copper wires were placed parallel to the platinum electrodes in each well of the cell culture plate and then connected to an external potentiostat. The joints were fixed with PDMS adhesive. The electrodes were placed perpendicular to the conductive hydrogel with a 2 cm spacing. The potentiostat sets the input voltage, providing an equal voltage for each well. The electrodes within each aperture were connected in parallel to ensure a uniform output voltage distribution^214^(Fig. 8e, f).

**Fig. 9:**
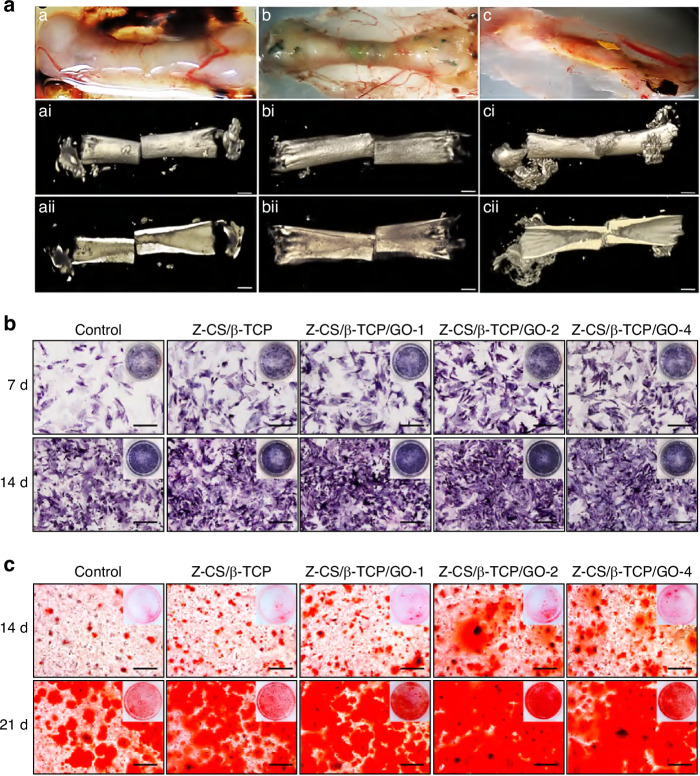
The effect of electrical hydrogels on stimulating bone regeneration. **a** Repair chicken bone defects using bovine ECM hydrogel and biocompatible mPCL scaffolds in the chick embryo CAM model. c, c1, c2: New bone formation and bridging were observed in the Stro-4+ seeded ECM/mPCL scaffold group.^233^ Copyright 2020, Wiley-VCH. **b**, **c** Osteogenic activity of BMSCs in Z - CS/β - TCP/GO hydrogels. ALP strain staining of BMSCs cultured with different scaffolds for 7 and 14 days, scale bar, 200 μm (**b**). Alizarin red S staining of cells cultured with varying scaffolds for 14 and 21 days, scale bar, 200 μm (**c**).^234^ Copyright 2023, MDPI. ECM extracellular matrix, mPCL melt electro-written medical-grade polycaprolactone, CAM chorioallantoic membrane, ALP alkaline phosphatase

When ES acts directly on cells, it influences a series of behaviors and functions of the cells. Specifically, ES can activate intracellular signal pathways such as PI3K/AKT and MEK/ERK, which play a crucial role in bone cell growth, differentiation, and survival.^153,175,181,211^ By regulating these signal pathways, electrical hydrogels can promote the osteogenic differentiation of bone cells and inhibit the activity of osteoclasts, thereby maintaining the normal metabolism and repair of bone tissue. Meanwhile, the electrical signals generated by hydrogels attract and guide stem cells, osteoblasts, and other cells to migrate toward the site of the bone defect, promoting their proliferation (Fig. 9b, c). This helps to increase the number of cells in the bone repair area and accelerate the formation of new bone.^195,208^ Additionally, ES can promote intercellular communication and interaction, as well as coordinate the functions of different cell types involved in bone regeneration. For example, it can enhance the connection between osteoblasts and vascular endothelial cells, promoting angiogenesis and providing a sufficient supply of nutrition and oxygen for new bone tissue. Conductive hydrogels can also stimulate bone regeneration by regulating the physical and chemical properties of the local microenvironment, such as pH value and ion concentration. For example, they can release bioactive ions, such as calcium and phosphate ions, which are essential in mineralizing and repairing bone tissue.^194,202^

These effects have enabled hydrogels responsive to electric field stimulation to be effectively applied in the treatment of bone defects, osteoporosis, and fractures. For example, compared to conventional hydrogels made by mixing chondroitin sulfate with gelatin (GO), hydrogels incorporating piezoelectric nanomaterials can induce lattice deformation under ultrasonic pressure, generating a potential difference and thereby depolarizing the cell membrane. Based on this, the osteogenic differentiation capacity of KBGO hydrogels was enhanced. In a rat cranial defect model, the bone volume-to-tissue volume ratio (BV/TV) and bone mineral density (BMD) at 6 and 12 weeks were significantly higher than those in the control group. Immunohistochemistry revealed abundant expression of the osteogenic markers Runx2 and OCN (Fig. 9d, e). Table 3 summarizes the cellular responses, signal pathways, and animal models of various electric field stimulation-responsive hydrogels.

**Table 3:** The effect of electrical hydrogels on stimulating bone regeneration

Hydrogel	Cellular effect	Signaling pathway/ gene transcription	Animal model	Ref.
CaP-PEDOT:PSS-MgTiO3@MA	BMSC differentiation toward osteogenesis	↑TGF-β/Smad2	Rat femur fracture model	^194^
GelMA-PPy-Fe	BMSC differentiation toward osteogenesis, macrophage polarization	↑Notch/MAPK/SMAD ↓Wnt/β-Catenin	-	^153^
GelMA-BP@Mg	Antimicrobial resistance, BMSC differentiation toward osteogenesis, and neurogenesis	-	Rat-infected cranial defect model	^180^
GelMA-BP	Vessel formation, macrophage polarization towards anti-inflammation, and DPSC osteogenesis	-	Rat mandibular defect model	^213^
GelMA/PPy	BMSC differentiation toward osteogenesis	↑Notch, BMP/Smad, calcium signaling pathway	-	^214^
CNT/CMC/BMP2 scaffold	hADSC osteogenic differentiation	↑Osteogenesis-related proteins(Col1A1,Runx2,OPN,BSP,OSX,OCN)	Rat cranial defect model	^181^
PVA/CH/SA hybrid scaffolds	MG63 Osteoblast Viability Enhancement, Antibacterial Resistance	-	-	^147^
Cel - PVA - Si	Increased MG63 cell adhesion, survival, and proliferation	-	-	^179^
Mo2Ti2C3 MXene hydrogel	neurogenesis, osteogenesis	↑ nerve growth factor, brain-derived neurotrophic factor	Mouse cranial defect model	^235^
GO-PAA	BMSC differentiation into neurons, cell proliferation, and adhesion	-		^236^
BaG/Gel/PEDOT: PSS scaffolds	Cell proliferation and metabolism	-	-	^237^
GelMA-BG-MWCNT nanocomposite hydrogel	BMSC cell adhesion and proliferation	↑Sp7, Spp1, BPS	-	^238,239^
BP-CNTpega-Gel	Cell adhesion and proliferation, osteogenic differentiation of pre-osteoblast cells.	↑CSF-1, GDF10,VEGFA,Smad1,CSF3,NOG and ITGB1	Rabbit femoral defect model	^244^
G-OP, GP5-OP, GP10-OP, GP15-OP	Cell adhesion, sustained release of oxygen delivery	-	-	^240^
MXene/RSF hydrogel	BMSC differentiation towards osteoblasts, macrophages polarization to M2 type, angiogenesis	↑RUNX-2, COL-1, CALM	Rat cranial defect model	^187^
ECM-AlgMA-BP@PDA hydrogel	BMSC differentiation towards osteoblasts	↑PI3K/AKT, Wnt, MAPK ↑PIEZO1, PIEZO2	Rat femur fracture model	^211^
dECM-P Gel-PC	BMSC differentiation towards osteoblasts	↑PI3K/Akt	Parma porcine osteochondral defect model Rabbit knee joint model	^202^
Cs/Gel/PHA/PBT hydrogels	macrophages polarization to the M2 type and angiogenesis	↑PI3K/Akt	Rat cranial defect model	^194^
NF-sPLLA	ADSC differentiation to chondrocytes	↑TGF-β1	Rabbit cartilage defect model	^208^
KBGO hydrogel	BMSC differentiation towards osteoblasts, macrophages polarization to M2 type	↑PI3K/AKT ↑MEK/ERK ↑calcium signaling pathway	Rat cranial defect model	^195^
GelMA and PiezoGEL hydrogels	Antimicrobial resistance, BMSC differentiation toward osteogenesis, and ECM mineralization	↑oxyR	Mouse periodontitis model	^241^
CoFe2O4/GGMA/PVDF	Simulation of bioelectrical environment	-	-	^242^
Bio-S	osteogenesis, angiogenesis, and neurogenesis	↑Runx2, VEGF, nestin, TUBB3	Rat cranial defect model	^243^
GelMA+c-BTO GelMA+t-BTO	macrophages polarization to M2, osteogenic differentiation of PDLSCs	↑phosphatidylinositol signaling, inositol phosphate metabolism, OXPHOS, and TCA cycle ↓IL-1 β,IL-6,CCL2,SOD2	Rat periodontal defect model	^220^

### Biomimetic simulation of extracellular matrix

Hydrogels are polymer networks with high water content and excellent biocompatibility, whose structure is highly similar to that of the ECM. They can reconstruct the local microenvironment required for bone repair from multiple dimensions.

First, hydrogels can mimic the natural mechanical microenvironment. Hydrogel scaffolds with a certain degree of stiffness can simulate the mechanical environment of the natural bone tissue ECM, providing stable physical support for bone cells, maintaining the spatial structure of the bone defect site, and preventing collapse of surrounding tissues.^195^ Adjusting the stiffness of the hydrogel scaffold can guide the ordered activity of cells at the bone defect site, promoting bone tissue repair and regeneration (Fig. 9a).^202^ D. Pasqui et al. innovatively added hydroxyapatite to carboxymethyl cellulose (CMC)-based hydrogels to mimic the inorganic-organic composite components in natural bone matrix, thereby enhancing cell proliferation and metabolic activity, and promote ECM mineralization^215^; introducing hyaluronic acid into collagen hydrogels not only improves scaffold mechanical stability but also recruits osteoprogenitor cells to participate in bone remodeling processes.^216,217^

Secondly, by optimizing the mechanical microenvironment, stable stress transmission is provided. Cui et al. developed piezoelectric composite hydrogel microspheres (containing barium titanate and polypyrrole), where the piezoelectric barium titanate enables stress-to-electricity conversion, and polypyrrole reduces energy loss during stress-to-electricity conversion. This material reduces tissue-to-tissue transduction losses by minimizing losses in the stress-to-electricity conversion process, thereby decreasing cartilage damage.^159^

Finally, hydrogels can locally regulate the inflammatory microenvironment. For pathological scenarios such as diabetic periodontitis, Liu et al. designed a polyphenol-mediated alginate/gelatin hydrogel, which forms a dual antioxidant system through the catechol structure of polydopamine and the sustained release of hydrogen sulfide from bovine serum albumin nanoparticles: polydopamine can scavenge reactive oxygen species, improving oxidative stress induced by hyperglycemia; the sustained release of hydrogen sulfide recruits MSCs, promotes angiogenesis and osteogenic differentiation, and alleviates inflammatory responses by regulating macrophage polarization.^218^

This microenvironment regulation mimicking the ECM differs fundamentally from the drug delivery systems discussed later, although hydrogels often require the incorporation of drugs or molecules to optimize the microenvironment. The core of microenvironment regulation lies in restructuring the physical, chemical, and biological microenvironment of the ECM through the inherent properties of the material, thereby passively regulating cellular behavior. The material directly interacts with the tissue microenvironment (e.g., providing mechanical support, regulating redox states). In contrast, drug delivery systems rely on carriers to transport and control the release of active molecules, actively intervening in physiological processes through molecular signaling pathways or pharmacological effects to mediate biological effects.

### Precise delivery or controlled release of active molecules and drugs

Due to their three-dimensional network and porous structure, hydrogels have become an ideal carrier for precise drug delivery.^219^ Through surface mineralization, shell modification,^220^ or optimization of electrostatic interactions, controlled drug release can be achieved. Temperature-sensitive hydrogels regulate release kinetics through their phase transition behavior, conferring antimicrobial and osteogenic therapeutic functions.^19,212^In bone-forming factor delivery systems, such as those represented by BMP-2, hydrogels improve BMD and trabecular number (Tb.N) in animal models by protecting protein activity and maintaining local concentration.^181,219^

Recent drug delivery systems emphasize the functional integration of hydrogel drug delivery systems of multiple therapeutic modalities. For example, the introduction of metal ions simultaneously achieves conductivity and antimicrobial properties. Mg^2+^ disrupts cell membrane permeability by binding to bacterial sulfhydryl groups,^180^ while CNTs inhibit bacteria through multiple mechanisms, including membrane damage and oxidative stress.^147^ Additionally, the antibacterial activity of carbon nanomaterials may be influenced by their composition, surface concentration, size, shape, number of layers, and surface functionalization. The synergistic design of such antimicrobial components with drug delivery has also become a research hotspot in recent years. Additionally, the electroactive gelatin methacrylate hydrogel developed by Xie et al. gains conductivity through a tannic acid-mediated polypyrrole microfiber network and utilizes a metal-polyphenol network to encapsulate exosomes. In a rat bone defect model, this system simultaneously regulates the inflammatory microenvironment (tannic acid scavenges reactive oxygen species) and induces osteogenesis (exosomes promote osteogenic differentiation). Electrical stimulation further enhances antioxidant persistence by maintaining the quinone-catechol redox cycle. This multifunctional hydrogel establishes an electroactive-bioactive coupled system, providing a novel strategy for complex bone healing scenarios.^221^ Wang et al. prepared GOMP hydrogels by loading doxorubicin into a dual network of gelatin methacrylate/oxidized dextran, combined with the photothermal conversion capability of polypyrrole (31.61% efficiency under 808 nm laser), achieving a chemotherapy-photothermal synergistic effect in osteosarcoma treatment; simultaneously, the incorporation of montmorillonite-strontium not only enhances the mechanical properties of the hydrogel but also promotes bone regeneration through Sr^2+^ release, demonstrating a “tumor clearance-bone repair” dual function, establishing a multi-functional system integrating photothermal therapy, chemotherapy, and bone regeneration.^160^ Mao et al. designed an oxidized graphene nanoplate/GelAMA composite hydrogel, using tannic acid as a functional molecule, which achieves stable loading through π-π stacking interactions—at the same time, promoting osteoblast differentiation, inducing angiogenesis through the paracrine effect of VEGF, establishing an osteogenic-vascularization-antimicrobial coupled system for repairing bone defects.^222^

Such studies integrate the multifunctional design of hydrogel matrices, combining electro-responsive, photothermal, and metal ion antimicrobial properties with drug delivery, thereby providing interdisciplinary solutions for complex clinical scenarios, such as infected bone defects and post-surgical tumor repair.

### Dynamic monitoring of bone tissue regeneration

In situ monitoring of bone regeneration can enable timely diagnosis and intervention by obtaining important biological parameters. However, there is a gap in effective methods for continuously and dynamically monitoring the process of bone tissue regeneration, including bone formation. EIS is an electrochemical testing technique that monitors real-time cell changes caused by proliferation, apoptosis, or cell-cell interactions.^223,224^ Evgeny Kozhevnikov’s team utilized this technique to develop a dual-conduction integrated biosensing system combining EIS and imaging techniques to study 3D cell culture for bone regeneration. Specifically, EIS reflects the interaction between cells and the electrode surface, the ion diffusion in the electrolyte around the cells, and the internal charge transfer within the cells by measuring the electrical properties between the electrodes and the electrolyte. When cells are adsorbed on the electrode surface, changes in the physiological state of the cells affect ion diffusion or changes in the internal charge distribution and transfer within the cells; the relevant parameters (such as resistance and capacitance) in the EIS spectrum will change. Fluorescence microscopy observes cells’ proliferation, migration, and other behaviors by detecting the fluorescent proteins expressed by the cells. The results of the two detection methods complement each other, thereby enabling comprehensive monitoring of the three-dimensional cell culture system^182^ (Fig. S3).

**Fig. 10:**
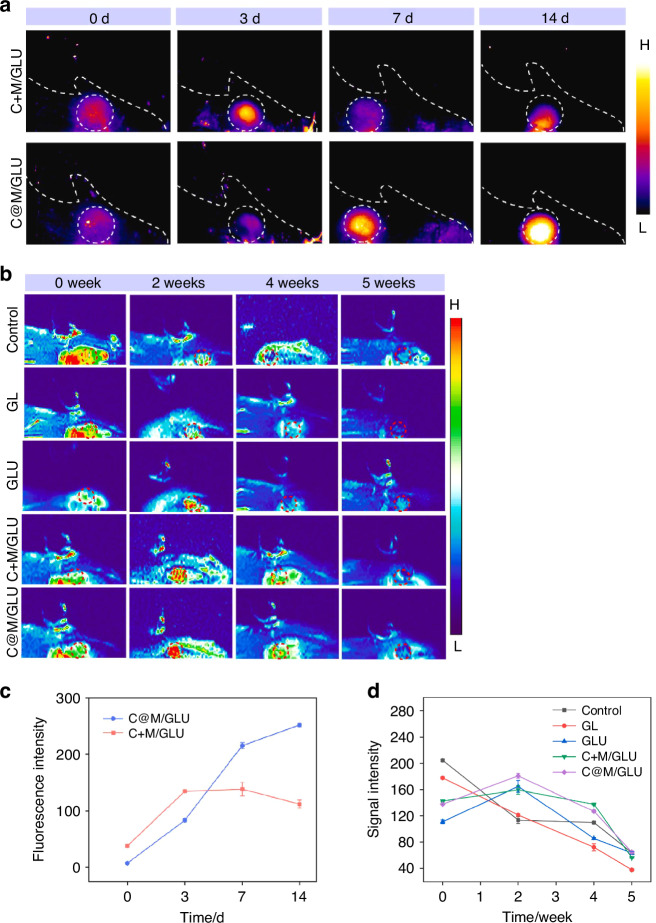
Electrical hydrogels can be used to monitor bone regeneration. **a**–**d** In vivo NIR-FL/MR imaging studies of different scaffolds through a rat calvarial defect model. NIR-FL images (**a**) and FL intensity curves (**c**) of ALP expression in the bone defect area at different times (λex = 660 nm). MR images (**b**) and signal intensity curves (**d**) for monitoring scaffold degradation.^225^ Copyright © 2024 Elsevier Ltd. LCR meter Inductance capacitance resistance meter, NIR-FL/MR near-infrared fluorescence/magnetic resonance imaging

During the process of bone regeneration, the activities of cells and changes in tissues lead to alterations in the local microenvironment, including changes in ion concentration and pH value. Yazhuo Huang’s team leveraged this characteristic to achieve non-invasive, real-time monitoring of bone repair by detecting changes in these electrochemical signals. They integrated CNT and BMP2 into chemically crosslinked CMC hydrogels to prepare a non-invasive and intelligent monitoring scaffold and used CV and EIS to monitor bone regeneration in real-time. Specifically, EIS curves were drawn on days 0, 3, and 7 after hADSCs were differentiated and cultured on the CNT/CMC scaffold—the smaller the arc diameter, the lower the charge transfer resistance. The cell differentiation situation can be evaluated by comparing the EIS curves at different times. Additionally, a cranial defect model was constructed in 8-week-old male SD rats for animal experiments. The scaffolds were implanted into the defects, and the rats were sacrificed at various time points. The scaffolds were then removed for CV and EIS tests to monitor the electrochemical responses of the CNT/CMC scaffolds in vivo, allowing for real-time monitoring of bone regeneration. However, at present, conductive hydrogels still face several challenges in the real-time tracking of bone regeneration, including the stability and specificity of signals, biocompatibility, and other issues. Future research needs to further optimize conductive hydrogels to improve monitoring accuracy and reliability, and promote their application in clinical bone regeneration treatment.^181^ ALP is also a marker that can reflect osteogenic activity. Feng’s team incorporated laponite, mesoporous silica loaded with Cyp (CyP@MSNs), and ultrasmall superparamagnetic iron oxide nanoparticles (USPIO@SiO2) into the bio-ink containing BMSCs and used bio-3D printing technology to prepare a functional scaffold C@M/GLU. CyP is a semicarbocyanine near-infrared fluorescence (NIR-FL) probe, interacts with alkaline phosphate (ALP) to emit fluorescence. This scaffold can be imaged using NIR-FL, which is beneficial for monitoring the expression of ALP and accurately reflecting the initial osteogenic activity of the scaffold. Meanwhile, the degradation of the scaffold can be monitored by tracking the changes in magnetic resonance signals at different time points. These findings indicate that the designed scaffold has the potential to serve as an in situ bone implant and can visualize osteogenesis and implant degradation throughout the bone repair process.^225^ (Fig. 10) In addition, labeling drugs or materials with fluorescein is also convenient for monitoring the position of drugs and the repair progress of the cartilage layer.^226^ However, the impact of fluorescein on biocompatibility, its toxicity, and degradability requires further study.

## Challenges and prospects

In summary, electrically stimulated hydrogels combine electrical stimulation as an adjunctive therapeutic modality with hydrogels, an emerging material, offering a highly promising comprehensive treatment strategy for bone regeneration in conditions such as traumatic bone injuries, osteoporotic fractures, and infectious bone defects. Compared to other treatment methods (such as autologous grafts and conventional hydrogels), electrically stimulated hydrogels offer the following advantages:

1. Precise adaptation to complex bone defect shapes. Unlike autologous grafts, hydrogels themselves are injectable and have self-adaptive deformation capabilities, avoiding the additional damage caused by the difficulty of shape matching and the need for secondary shaping in autologous grafts.
2. Controlled electrical stimulation for sustained activation. Unlike traditional methods that rely on exogenous growth factors (such as BMP-2), electrically stimulated hydrogels can continuously release physiological-level electrical signals through electrical effects, precisely regulating cell differentiation.
3. Synergistic multi-repair mechanisms. In addition to promoting bone regeneration through electrical stimulation, some systems also integrate antimicrobial, angiogenic, and natural bone simulation functions. These are difficult to achieve with autologous grafts or single-function hydrogels.
  However, the ultimate goal of biomaterial research is to translate clinical benefits to patients. Therefore, to further advance the clinical translation of these novel electrically responsive hydrogels, the following key bottlenecks still need to be addressed:
  1. Biological safety. Electrical materials inherently pose toxicity risks. These substances may enter the circulatory system through wounds, potentially causing adverse effects such as heavy metal accumulation or toxin buildup. Therefore, it is crucial to clarify the impact of these substances and their degradation products on the body. Current biological safety assessments are primarily limited to short-term toxicological evaluations, with insufficient assessment of the potential accumulation sites and quantities of nanomaterials in the body following long-term exposure, as well as their effects on physiological functions. Future research should focus on long-term biosafety and establish evaluation methods and indicators for adverse reactions such as electrophysiological disorders, chronic inflammation, or immune imbalance. Additionally, the drugs and components carried by electrical hydrogels may participate in molecular signal transduction and the metabolic cycling of organic substances. In this process, whether electrical materials, particularly certain metals and ions, affect normal tissues should be subject to more in-depth mechanistic studies, utilizing high-throughput sequencing technologies to comprehensively assess the role of these materials in tissue repair and regeneration.
  2. Animal models. In existing studies, rodent models are widely used. However, these small animal models typically exhibit strong self-healing tendencies. They cannot accurately simulate the complexity of human skeletal metabolism, which may result in therapeutic effects observed in rodents that are not fully applicable to human patients. Large animal models are indispensable for safety assessments, device testing, and modeling complex diseases before clinical translation, as their physiological structure, metabolic mechanisms, and long-term effects are more closely aligned with those of humans. Therefore, before advancing to clinical trials, it is essential to utilize larger animal models (such as pigs or monkeys) that more closely resemble human physiological conditions for further functional validation. However, while animal models provide a critical bridge to clinical translation, their essence remains a simplified representation of the human system. For the widespread clinical application of electrical hydrogels, their efficacy can only be validated in real patient populations through large-scale, multi-center, long-term follow-up clinical studies.
  3. Clinical feasibility. Although electroactive hydrogels appear to hold significant clinical potential, some conductive hydrogels require an external power source to generate local microcurrents, making transdermal power supply a considerable obstacle. Electromagnetic induction coupling results in energy attenuation as it passes through skin tissue, leading to a reduction in electrical signal strength. While built-in batteries provide a stable energy supply, they may pose risks such as chemical toxicity (e.g., battery electrolyte) and inflammatory reactions at the implantation site. Additionally, different stages of bone regeneration require dynamic adjustments of electrical stimulation parameters; however, existing systems lack real-time feedback, thereby increasing the patient’s burden of using external devices.

Therefore, future research should focus on optimizing and enhancing biosafety, selecting suitable animal models, and assessing clinical feasibility to promote the widespread clinical application of electrically stimulated hydrogels.

## Supplementary information

Supplementary Information
