# Supplementary material for: Electric field stimulation-responsive hydrogels for bone regeneration: from mechanisms to applications
Source: Bone Res. 2026 Jan 12;14:4. doi: 10.1038/s41413-025-00482-5 (PMC12791148; doi:10.1038/s41413-025-00482-5)
Supplement: Supplementary file 1 — Supplementary Information [file 41413_2025_482_MOESM1_ESM.docx]

**Supplementary Information**

**
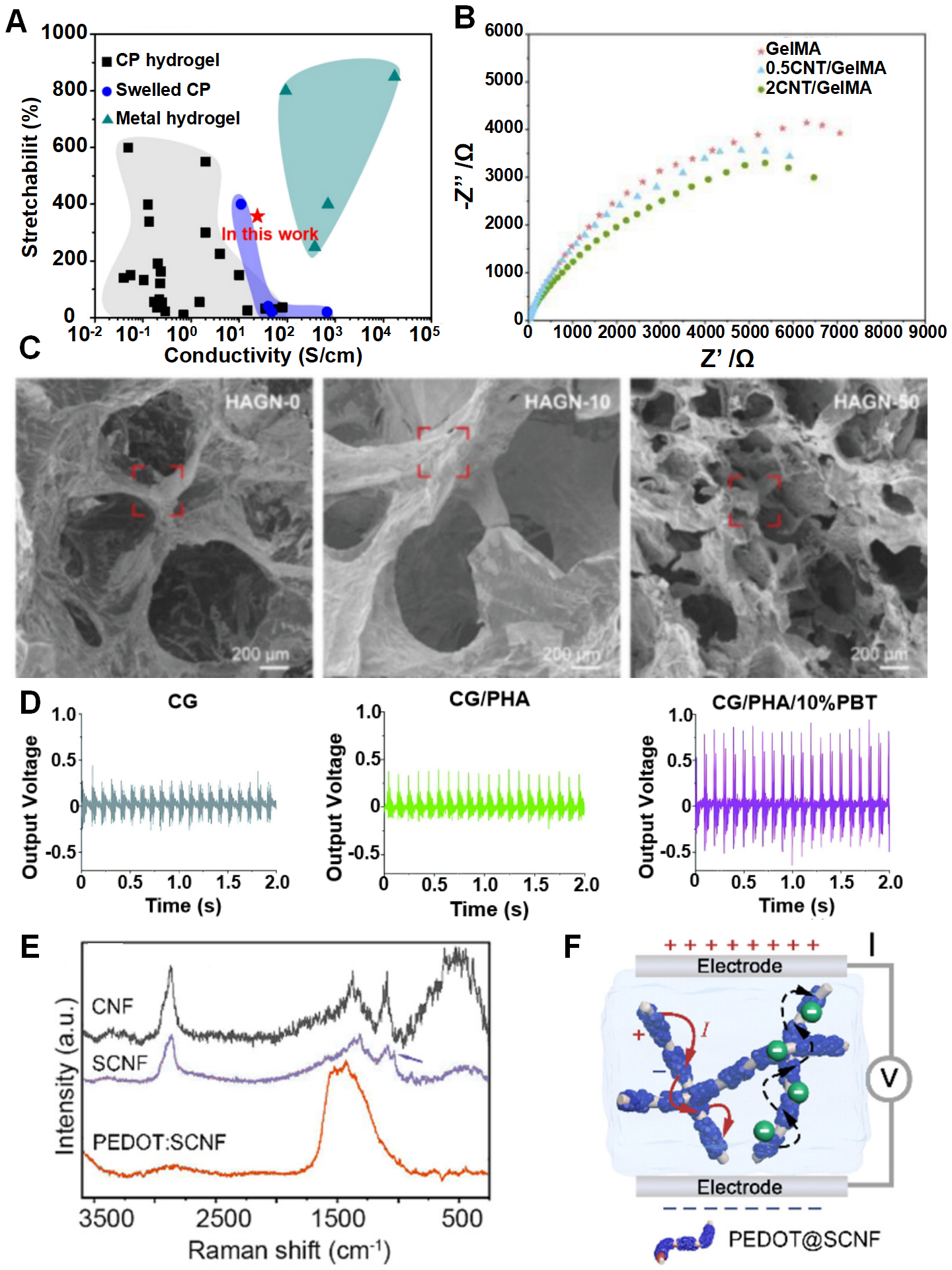
**

**Supplementary Figure 1.** (A) Conductivity-tensile property of the conductive hydrogel.^179^ Copyright 2024, American Association for the Advancement of Science. (B) Conductivity of the conductive hydrogel.^180^Copyright © 2024 Elsevier Ltd. (C) SEM images of HAGN hydrogels with different graphite concentrations (0%, 10%, 50%). ^181^ Copyright 2024, Wiley-VCH. (D) Dopamine-modified barium titanate participates in the crosslinking of hydrogels. Output voltages of different hydrogel samples under 10 Hz and 1 kPa pressure.^208^Copyright 2023, Springer Nature. (E, F)The combination of the drug and the hydrogel under electrostatic adsorption. (E) Raman spectrum. (F) Schematic illustration of possible electron/ion transport along the core sheath.230 Copyright 2024, American Chemical Society.


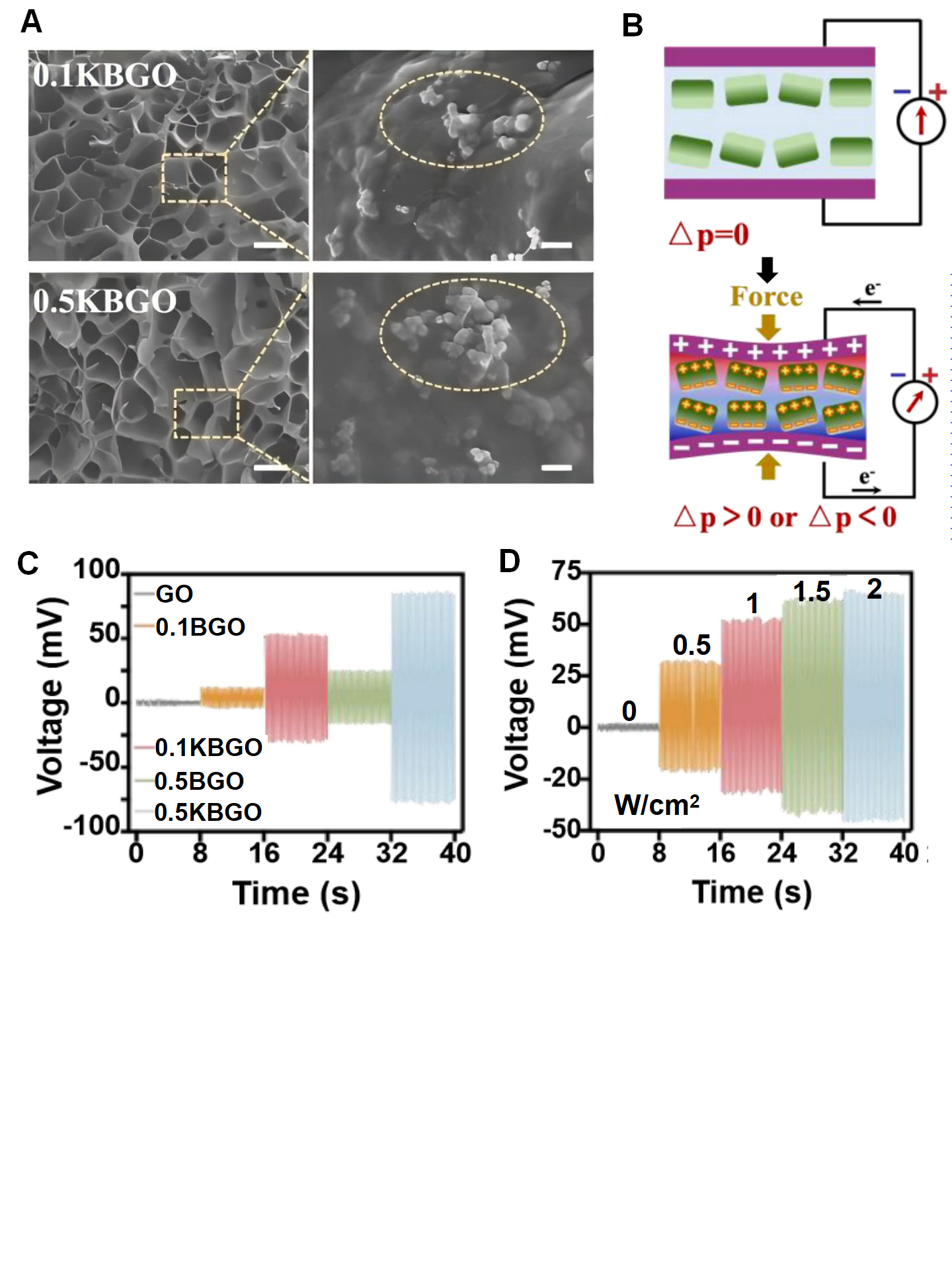


**Supplementary Figure 2.** (A - D) Amino-modified barium titanate participates in the crosslinking of hydrogels. (A) SEM images of GO, 0.1KBGO, and 0.5KBGO hydrogels and the embedded KBTO nanoparticles (ellipses with dotted borders). (B) Finite element simulation of the potential distribution of KBTO nanoparticles using COMSOL under the condition of with or without pressure generated by ultrasonic cavitation. (C, D) The output voltage of the 0.1KBGO hydrogel under the same-intensity ultrasound and an enlarged view under the stimulation of the sound-intensity ultrasound.^209^Copyright 2024, Springer Nature.


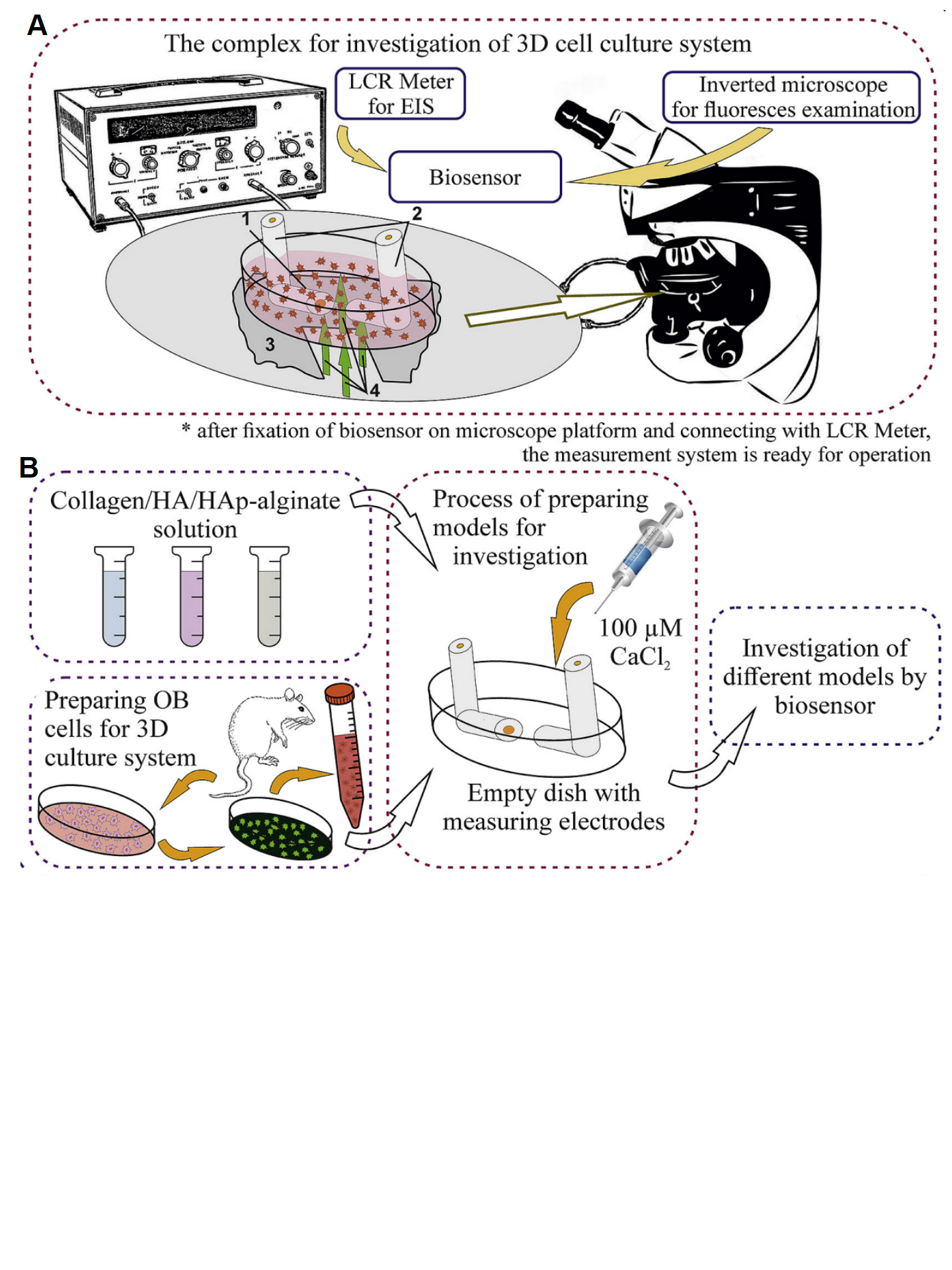


**Supplementary Figure 3.** (A) The schematic diagram of a dual-transduction integrated biosensing system mainly consists of an LCR meter for detecting impedance and an inverted microscope for detecting fluorescence. (B) 3D cell culture and its examination using the dual-transduction integrated biosensing system.

^187^Copyright © 2019 Elsevier Ltd.
